# Integrative taxonomy on the rare sky-island *Ligidium* species from southwest China (Isopoda, Oniscidea, Ligiidae)

**DOI:** 10.1186/s40850-022-00120-1

**Published:** 2022-05-23

**Authors:** Jin Wang, Jingbo Yang, Xuegang Zeng, Weichun Li

**Affiliations:** College of Agronomy, Jiangxi Agricultural University, Nanchang, 330045 China

**Keywords:** Geometric morphometrics, Molecular delimitation, Morphology, New species, Terrestrial isopods

## Abstract

**Background:**

The sky-island *Ligidium* species fauna in southwest China is poorly known. Before this study, six of the seven sky-island species of the genus were known to be endemic to southwest China. In morphology, *Ligidium* species are often difficult to identify, and an appraisal of integrative taxonomy is needed.

**Results:**

We integrated morphology and molecular analyses to delimit *Ligidium* species. Molecular species delimitation based on distance- and evolutionary models recovered seven-candidate lineages from five gene markers (COI, 12S rRNA, 18S rRNA, 28S rRNA and NAK). We also estimated that the species divergences of sky-island *Ligidium* in southwest China started in late Eocene (40.97 Mya) to middle Miocene (15.19 Mya).

Four new species (*L. duospinatum* Li, sp. nov., *L. acuminatum* Li, sp. nov., *L. rotundum* Li, sp. nov. and *L. tridentatum* Li, sp. nov.) are described. Morphological confusion among *L. denticulatum* Shen, 1949, *L. inerme* Nunomura & Xie, 2000 and *L. sichuanense* Nunomura, 2002 is clarified by integrative taxonomy.

**Conclusion:**

This work confirms that an integrative approach to *Ligidium* taxonomy is fundamental for objective classification, and deduced the uplift of Qinghai-Tibetan Plateau in the late Eocene and middle Miocene as one of the principal reasons for the species divergences of sky-island *Ligidium* in southwest China. We also inferred that sky-island mountains have a huge reserve of higher *Ligidium* species diversity.

**Supplementary Information:**

The online version contains supplementary material available at 10.1186/s40850-022-00120-1.

## Background

The terminology “sky islands” was first proposed to describe isolated mountain ranges in southeastern Arizona of America [1]. Unlike oceanic islands isolated by the sea, the high-elevation sky islands are separated by differing low-elevation habitats [2]. The complex topography and discontinuous landscape among the sky islands support diverse habitats and usually harbour high endemic species diversity, thus being noted as significant biodiversity hotspots [3, 4]. Also, additional related hypotheses, e.g., “species museum” and “species pumps”, were proposed based on sky-island organisms [5, 6]. Thus, it is important to explore species diversity in sky-island areas.

In China, the southwest region is composed of a series of discrete mountain ranges. It spans approximately N24°–N34° of latitude, including Chongqing City, Guizhou, Sichuan, Yunnan Provinces and Tibet Autonomous Region [2]. The mountains of southwest China and adjacent areas comprise one of the world’s biodiversity hotspots [3]. In this region, the high mountains are generally 2,000 m above sea level (asl) to 7,000 m (Mt. Gongga, 7,556 m asl) or higher [2]. Meanwhile, it is also characterized by complex geographic features, known as “sky islands” [7]. The unique alpine environment in southwest China has drawn the attention of many researchers, including various endemic alpine taxa [2, 4, 8, 9]. However, some groups of organisms, such as the genus *Ligidium* of terrestrial isopods (Suborder Oniscidea, Order Isopoda), are little known in the sky-island areas. In the records of high-altitude Isopoda in the Old World, only a single *Ligidium* species (*L. denticulatum* Shen, 1949) was mentioned [10].

**Fig. 1:**
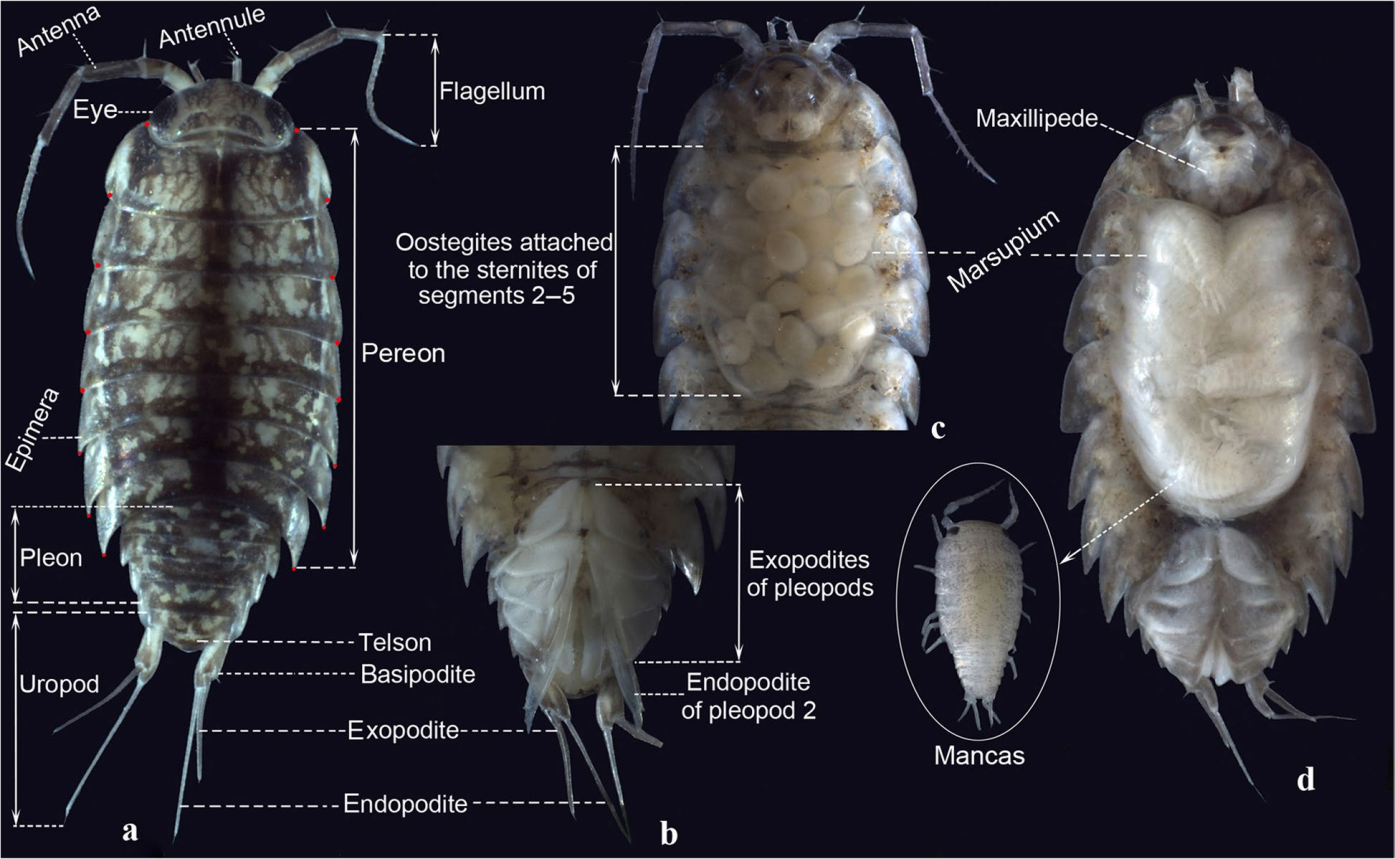
External morphology of a *Ligidium* sp. **a** Male adult on dorsal view; red circles showing landmarks to evaluate the variation of body shape in morphometric analysis. **b** Male pleon on ventral view. **c** Female with eggs in marsupium, ventral view. **d** Female with mancas in marsupium, ventral view

*Ligidium* is the most species-rich genus within the family Ligiidae (or the new family Ligidiidae proposed recently by Dimitriou et al. [11]). It was erected with *Ligidium persooni* Brandt, 1833 [a junior synonym of *L. hypnorum* (Cuvier, 1792)] as the type species [12]. *Ligidium* species usually have an elliptic dark grey body and bear pale muscle spots on the dorsal surface (Fig. 1a). In external morphology (Fig. 1a, b), they exhibit two well-developed eyes with many ommatidia, an antennule that projects beyond the front of the head, a flagellum of antenna with 7–23 segments, a well-defined sulcus at the posterior part of cephalon, reminiscent of the fusion with first ancestral thoracic metamere, pereonites much broader than the abruptly contracted pleon, a distal margin of the telson generally convex in the middle, a gently concave near lateral margin forming two blunted rounded corners, an uropod protopodite about twice as long as broad and of a unique hook-like shape, and an uropod endopodite more or less longer than exopodite. Furthermore, regarding sexual differentiation, males have a long and thin pleopod 2 endopodite (Fig. 1b, in females like in Fig. 1d), and ovigerous females bear eggs or juveniles in an ‘open’ marsupium (Fig. 1c, d). However, among morphological characters, only the structure of male pleopod 2 endopodite provides a relatively robust diagnosis at species level [13, 14]. Nevertheless, even this character may exhibit only minor interspecific differences among closely related species, causing difficulties in cryptic species exploration. Morphological variation of the apical part of the male pleopod 2 endopodites can cause additional confusion in some species recognition.

At present, a total of 54 *Ligidium* species are recognised mainly based on morphology, all of them distributed to the north of the Tropic of Cancer [15–35]. Among them, seven species were reported from China’s sky-island region. Six sky-island species (*L. denticulatum* Shen, 1949, *L. inerme* Nunomura & Xie, 2000, *L. jiuzhai* Tang & Zhou, 1999, *L. mimense* Nunomura & Xie, 2000, *L. sichuanense* Nunomura, 2002 and *L. watanabei* Nunomura, 2002) are endemic to southwest China. This could suggest that the unique sky-island environment of southwest China harbours an exceptional *Ligidium* fauna and more species of the genus may remain to be revealed.

In this context, we study *Ligidium* specimens collected from the sky islands of southwest China between 2012 and 2020. We aim to reveal new sky-island species, to delimit species by integrating morphology and molecular approaches, and to date speciation events of the sky-island *Ligidium* spp. in southwest China. In addition, we describe four new species.

## Materials and methods

### Sampling collection, morphologic preparation and identification

We fixed and stored the specimens in absolute ethanol and mounted the dissected appendages in a neutral balsam mounting medium. All studied specimens are deposited in the Insect Museum, Jiangxi Agricultural University, Nanchang, China (JXAUM).

Photographs were taken with a digital camera Zeiss AxioCam Icc 5 attached to a digital microscope Zeiss Stereo Discovery V12. Line drawings were made by the GNU Image Manipulation Program [36]. Species identifications were based on traditional morphological criteria. Terminology of morphology follows Schmidt [37].

For morphometric analysis, we selected 42 specimens representing seven sky-island *Ligidium* species of southwest China that were revealed by molecular delimitation. To evaluate variation in body shape, 16 landmarks were placed on pereonites 1–7 (Fig. 1a). The images were converted into TPS format using tpsUtil 1.56 [38]. Landmarks for all images were aligned, rotated and scaled using Procrustes superimposition [39]. Afterwards, we recorded the landmarks using TPSdig 2.17 [40] and analysed the data with MorphoJ [41]. To visualize shape variations across morphospace, we conducted principal component analyses (PCAs) and canonical variate analyses (CVAs). Mahalanobis and Procrustes distances were applied to evaluate the morphological variations among species.

### PCR amplification and sequencing

Genomic DNA was extracted from pereopods or pereonites of individuals using the TaKaRa MiniBEST Universal Genomic DNA Extraction Kit following the animal tissue protocol. Parts of two mitochondrial loci, cytochrome *c* oxidase I (COI) and 12S ribosomal RNA, and of the nuclear protein-coding genes Sodium-otassium Pump (NAK), along with 18S and 28S ribosomal RNA genes were amplified using polymerase chain reaction (PCR). Fragments of COI and 12S rRNA mtDNA genes were amplified using the primers LCO1490/HCO2198 [42] and 12SCRF/12SCRR [43, 44]. The primers pairs 18sai/18sbi, 28sa/28sb [45], NAK for-b/NAK rev2 [46] were used for the amplification of 18S rRNA, 28S rRNA and NAK respectively. PCR amplifications were performed with an initial denaturation at 95 °C for 3 min., followed by 35 cycles of 40 s. at 94 °C, 40 s. at 48 − 54 °C, and 40 s. at 72 °C, with a final extension at 72 °C for 10 min. The PCR products were sequenced by using an ABI3730XL DNA Analyzer (Applied Biosystems). All sequences were deposited in DDBJ (DNA Data Bank of Japan), with accession numbers listed in the Supporting Information (provided as Additional file 1).

### Phylogenetic analyses

The resulting forward and reverse sequences were assembled in SeqMan, manually checked for errors, searched with Blast to expose contaminants, and aligned using MAFFT 7.313 [47]. Aligned partitions of all five loci, mitochondrial genes-only (COI and 12S), nuclear genes-only (18S, 28S and NAK) and all of the datasets (COI, 12S, 18S, 28S and NAK) were concatenated into nexus files for downstream analyses. Due to a large amount of missing data, the sample BJS2011 was excluded from the mtDNA-only datasets.

We conducted Bayesian inference (BI) and Maximum likelihood (ML) analyses in three separate analyses in which the data were partitioned as follows: (i) by two mitochondrial genes (COI and 12S); (ii) by three nuclear genes (18S, 28S and NAK); (iii) by five concatenated genes (COI, 12S, 18S, 28S and NAK). The BI analyses were conducted in MrBayes 3.2.6 [48] on the platform of PhyloSuite [49]. Four chains of Markov chain Monte Carlo (MCMC) were run simultaneously for a total of 3 million generations. The following criteria were used to determine if the Bayesian analyses had reached convergence: (i) the posterior probability values tended to be stable; (ii) the average standard deviation of the split frequencies of independent runs became stable and approached zero; (iii) potential scale reduction factor was close to one; (iv) the Effective Sample Size (ESS) for the posterior probabilities evaluated in Tracer v.1.7 [50] exceeded 200. The sampling frequency was set to 100. The number of runs and burn-in fraction with 2 and 0.25, respectively. The proportion of trees that contained the clade was given as the posterior probability (PP) on the consensus tree to estimate the robustness of each clade. ModelFinder [51] was used during these analyses to set appropriate models of sequence evolution for each partition under the Akaike information criterion. HKY + F + I + G4, HKY + F + G4, GTR + F + G4, GTR + F + G4, SYM + G4 was selected for COI, 12S, 18S, 28S and NAK, respectively. The ML trees were constructed on the platform of PhyloSuite [49] with 1000 bootstrap replicates using IQ-TREE 2 [52]. *Armadillidium vulgare*, *Porcellionides pruinosus*, *Styloniscus* sp., *Spherillo dorsalis* and *Spherillo obscurus* were used as outgroups for phylogenetic reconstructions (GenBank accession nos. provided as Additional file 1). The resulting gene phylogenies were visualized in FigTree 1.4.3 [53]. TCS 1.21 [54] was used to generate a haplotype network based on the concatenated datasets of three nuclear genes (18S, 28S and NAK) using statistical parsimony [55] available in PopART 1.7 [56].

### Molecular species delimitations

A single mitochondrial marker, 12S, was employed to generate initial species hypotheses. Based on the 12S sequences, automatic barcode gap discovery (ABGD) automatically clustered sequences into candidate species by detecting barcoding gaps using a command-line version with the default parameters [57].

Furthermore, Bayesian implementation of the Poisson tree processes model for species delimitation (bPTP) [58] and general mixed Yule coalescent (GMYC) model [59, 60] were used to assess the support for initial groupings of ABGD. The bPTP model was performed with the default parameters [58]. The input species tree was estimated using IQ-TREE 2 [52] with 12S data alone. The GMYC identified the input species tree was estimated using BEAST 2 [61] based on 12S data, and the best-fitting substitution model was assessed using ModelFinder with TN + F + G4 [51]. We selected the relaxed molecular clock model in the trial operation based on the reliable evidence, i.e., absolute value of ML1-ML2 is more than twice the sum of the SDs (ML1 = -3919.17, SD1 = 14.93, ML2 = -3841.81, SD2 = 13.42 in the relaxed, strict clock log-normal models; BF = 78.17) [62, 63]. The run was executed twice for 5 million generations and 10 burn-in percentage.

Finally, we analysed the multilocus data (COI, 12S, 18S, 28S and NAK) under the multispecies coalescent model to delimit species with BPP 4.4 [64]. We integrated the previous consensus results to specify the guide trees. Each analysis was repeated twice used algorithm 0 with the “joint species delimitation and species-tree inference or unguided species delimitation (A11)”, 200,000 generations with the first 20,000 burn-in and the samples were taken every five generations. We assumed three combination priors about the population size parameters (θs) and the divergence time at the root of the species tree (τ0): (i) large population sizes θ ~ IG (3, 0.2) with mean 0.2/(3–1) = 0.1 and deep divergences τ0 ~ IG (3, 0.2) with mean 0.1; (ii) large population sizes θ ~ IG (3, 0.2) with mean 0.2/(3–1) = 0.1 and shallow divergences τ0 ~ IG (3, 0.002) with mean 0.001; (iii) small population sizes θ ~ IG (3, 0.002) with mean 0.002/(3–1) = 0.001 and shallow divergences τ0 ~ IG (3, 0.002) with mean 0.001 [65], other divergence time parameters were specified by the uniform Dirichlet distribution [66].

### Divergence time estimation

Species tree and divergence time were estimated based on the COI dataset, including five Chinese sky-island species and five European species of *Ligidium* (DDBJ/GenBank accession nos. provided as Additional file 1). *Ligidium rotundum* and *L. inerme* were excluded from this estimation due to a large amount of missing data. Specimens were assigned to species a priori by the result of BPP above. The best-fitting substitution model was assessed in ModelFinder [51] with TN + F + G4. The hypothesis that our data evolved according to a relaxed molecular clock model was selected by the absolute value of ML1-ML2 that is more than twice the sum of the SDs (ML1 = -4486.54, SD1 = 2.00, ML2 = -4494.61, SD2 = 1.94 in the relaxed, strict clock log-normal models; BF = 8) [62, 63]. The crustacean mitochondrial COI clock of 0.0115 substitutions per site per million years was applied as calibration [67–69]. The analysis was carried out in BEAST 2 [61] under an uncorrelated lognormal relaxed molecular model with nested sampling and Yule speciation prior. The run was executed twice for 10 million generations and 10 burn-in percentage.

### Distribution mapping

The distribution map across southwest China was made using DIVA GIS 7.5 based upon topographic grids were retrieved from the WorldClim database (http://www.worldclim.org) [70], the collection localities of the specimens examined in this study and the literature [27, 31, 33, 34] (provided as Additional file 2).

### Nomenclatural acts

The electronic edition of this article conforms to the requirements of the amended International Code of Zoological Nomenclature (ICZN), and hence the new species names contained herein are available under that Code from the electronic edition of this article.

## Results

### Morphological analyses

**Fig. 2:**
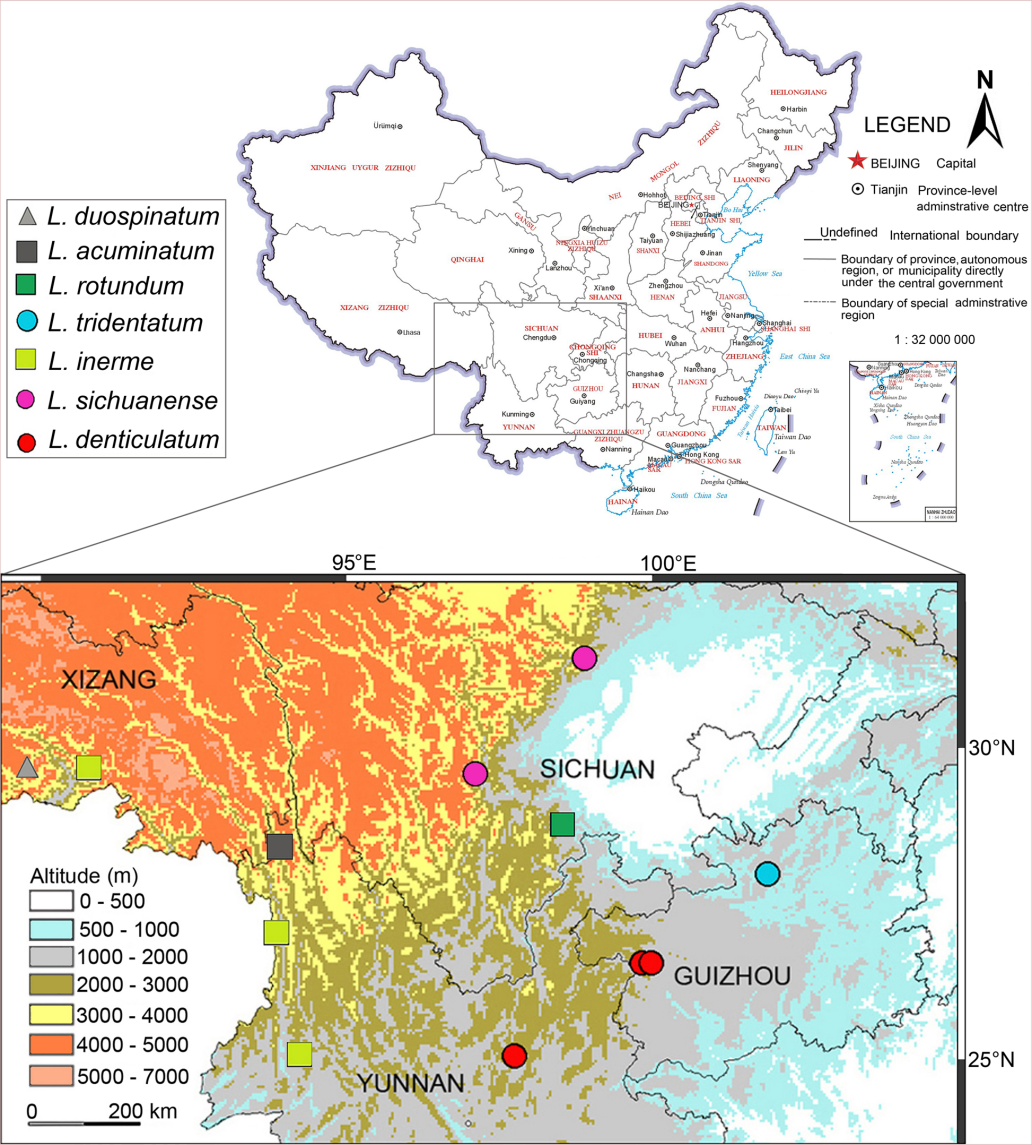
Map of the study area showing the recorded localities of the genus *Ligidium*

The morphological characters of the specimens collected from southwest China were analysed using appendages, including the antennule, antenna, mouthparts, pereopods and pleopods. As a result, seven species were preliminarily recognized, all of them distributed in the mid- or high mountainous areas (Fig. 2). A key to the sky-island *Ligidium* species is presented. Nevertheless, the taxonomic confusion among *L. denticulatum* Shen, 1949, *L. inerme* Nunomura & Xie, 2000 and *L. sichuanense* Nunomura, 2002 remained unclarified because of the minor differences in the form of their male pleopods 2. It is difficult to decide whether their minor differences are due to interspecific divergence or intraspecific variation, based on morphology alone.

**Fig. 3:**
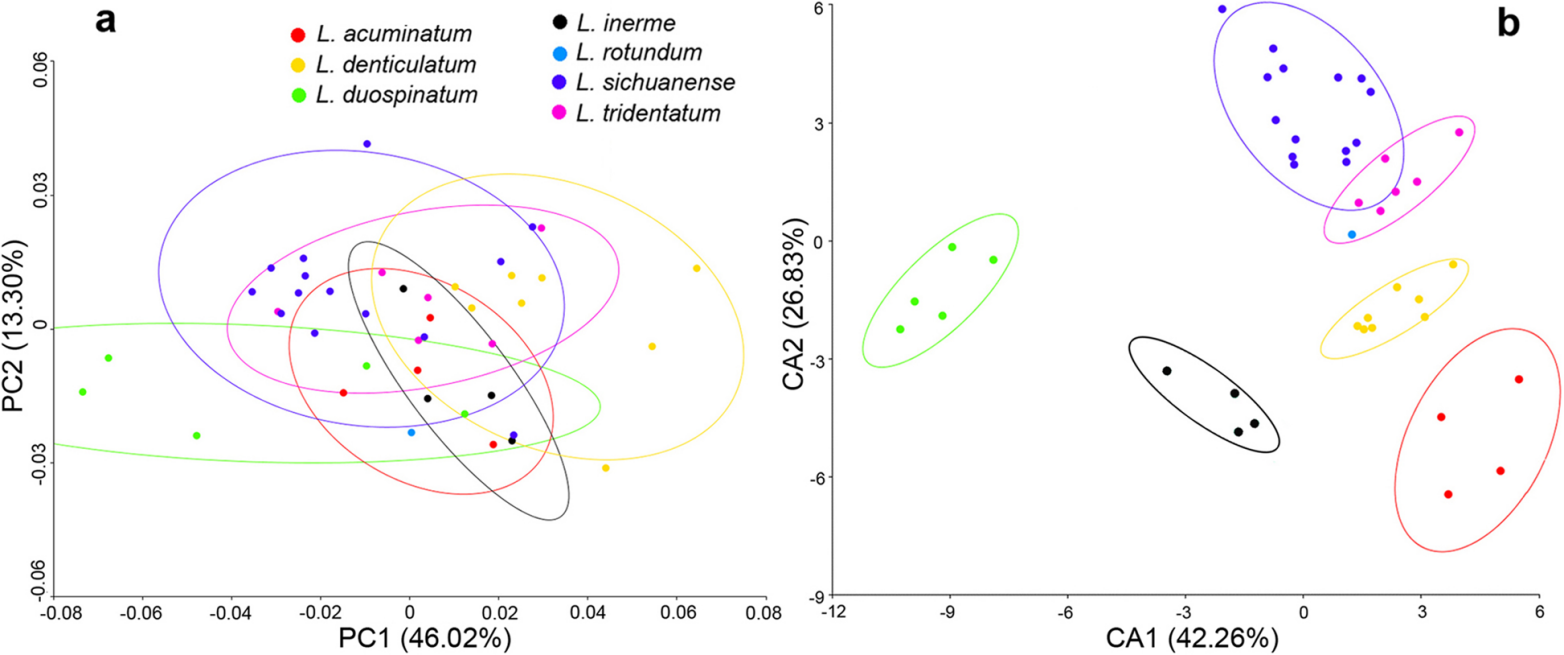
Scatter diagrams of geometric morphometrics. **a** Principal component analysis (PCA). **b** Canonical variate analysis (CVA)

In the analyses of geometric morphometrics, the error of manual sampling was assessed based on the images of *Ligidium acuminatum* Li, sp. nov. The data were obtained by repeated sampling ten times. Mahalanobis and Procrustes distances are insignificant (*p* > 0.05), indicating that the selection of landmarks in this context were precise. In morphological variation, we obtained a total of 28 principal components and six variables. The first principal component (PC1) takes up 46.02% of the total shape variation and showed an extensively overlapping distribution of species in morphospace (Fig. 3a); the CVA with the first two canonical variables (CV1 and CV2) can unambiguously classify all the species except for *L. rotundum* Li, sp. nov. (Fig. 3b), because only one individual of this species was available in a good condition for geometric morphometrics analysis, leading to limited data.

### Molecular analyses

**Fig. 4:**
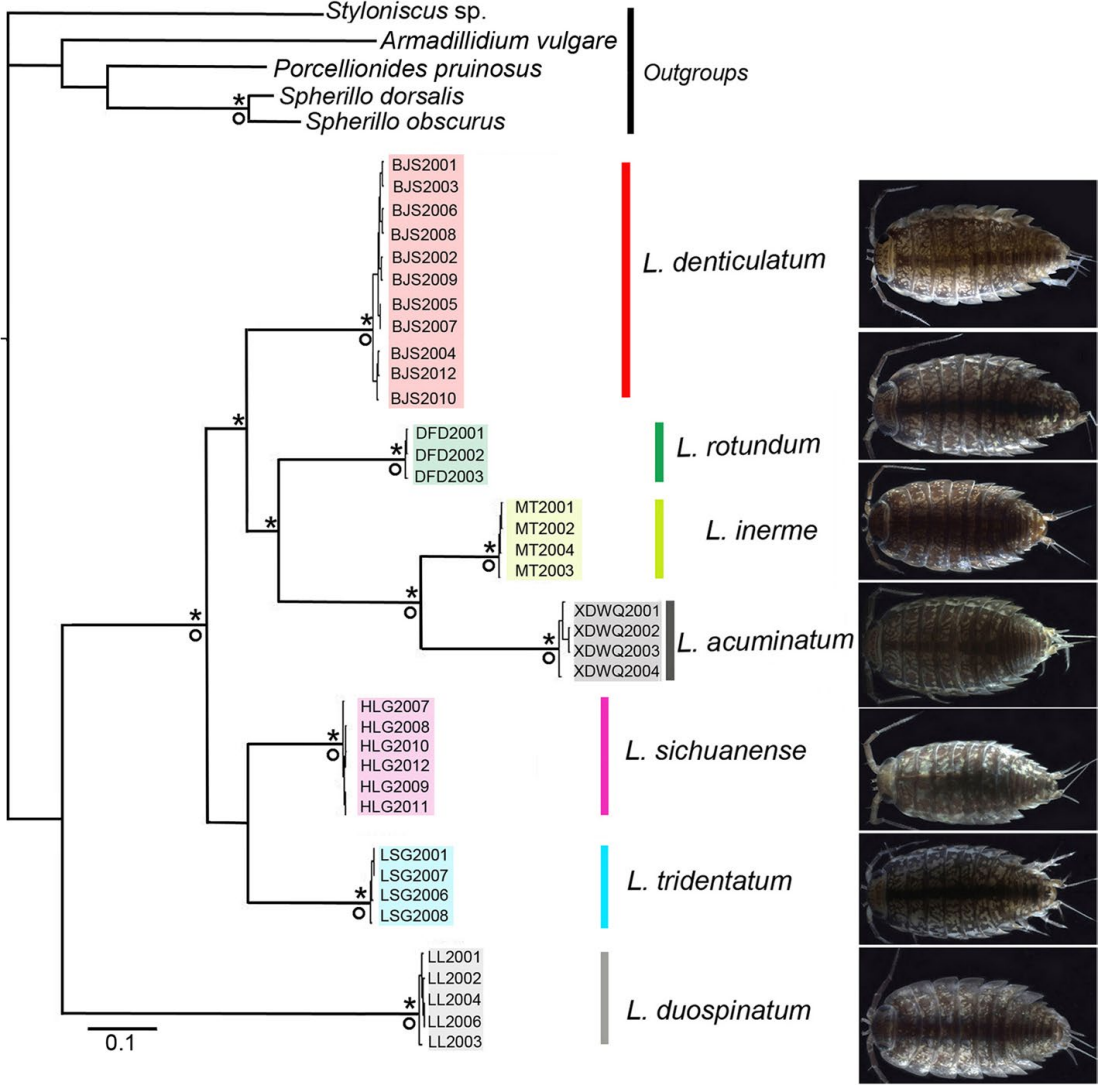
Bayesian consensus tree based on two mitochondrial genes (COI and 12S). Asterisks and circles represent high node support with Bayesian posterior probabilities > 0.95 and maximum bootstrap values > 95

**Fig. 5:**
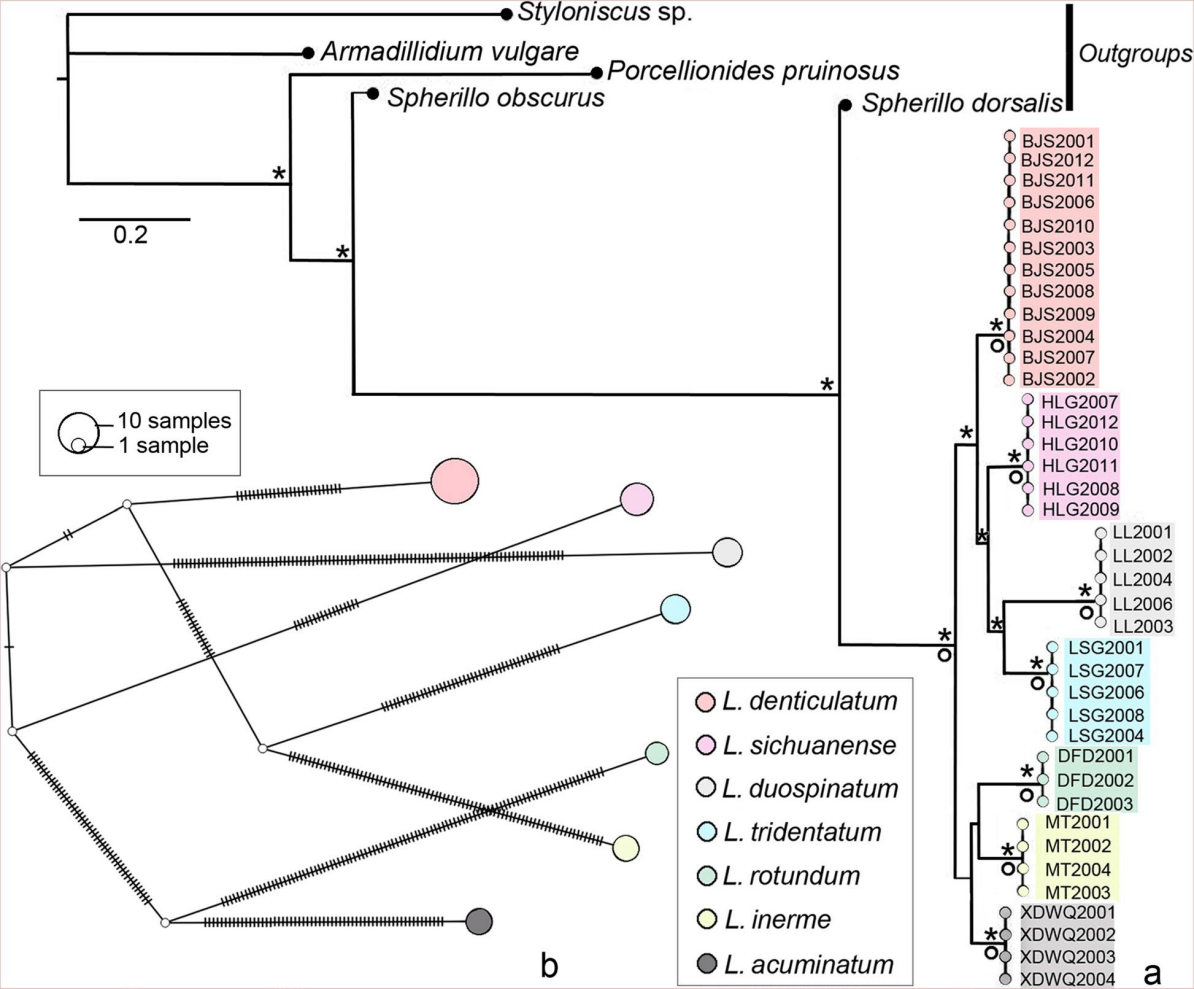
Bayesian consensus tree and haplotype network based on three nuclear genes (18S, 28S and NAK). **a** Bayesian consensus tree. Asterisks and circles represent high node support with Bayesian posterior probabilities > 0.95 and maximum bootstrap values > 95. **b** Haplotype network indicating the distribution of unique *Ligidium* haplotypes

**Fig. 6:**
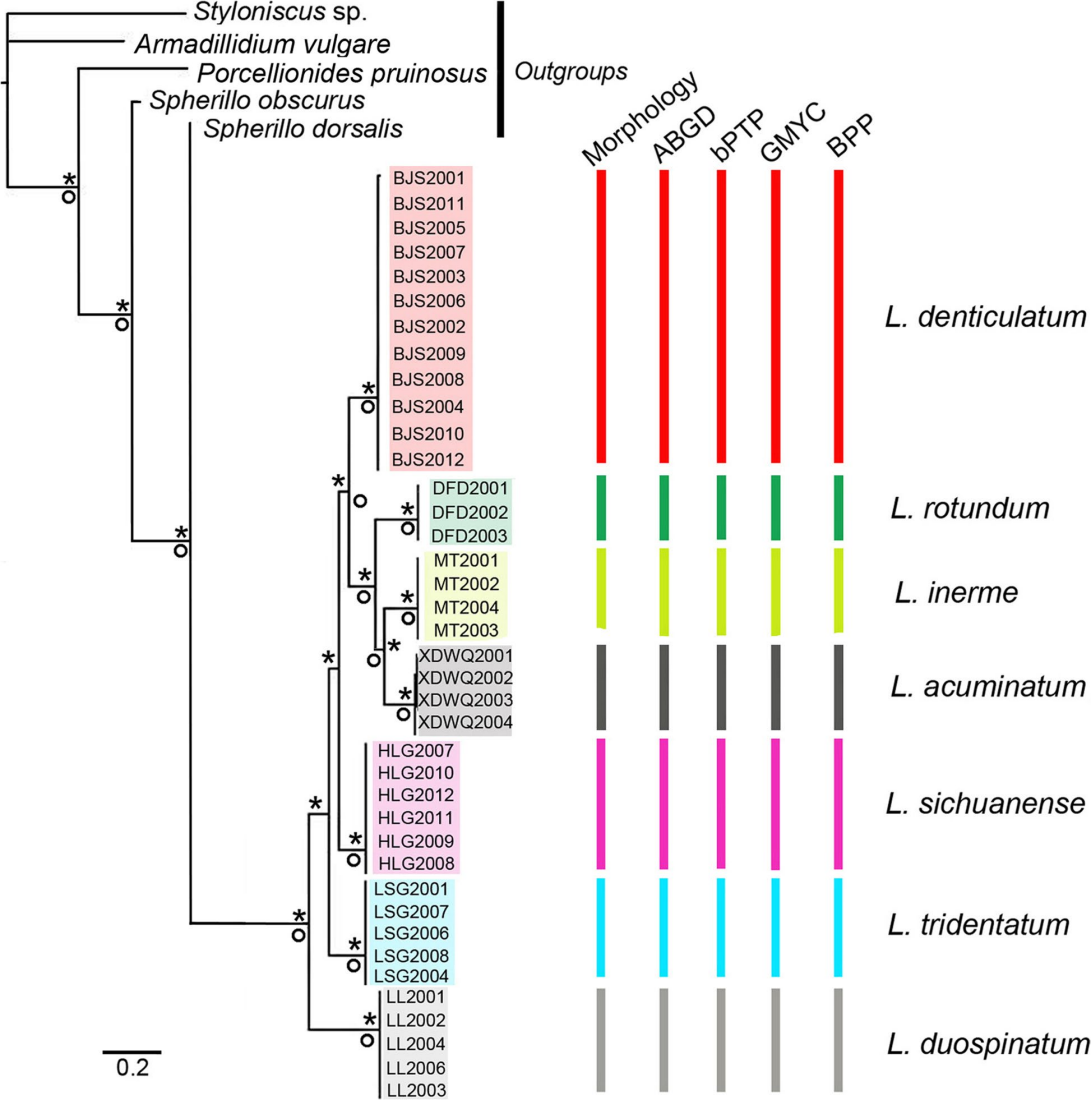
Species delimitations summarised on a Bayesian consensus tree based on a concatenated dataset of six loci (COI, 12S, 18S, 28S and NAK). Asterisks and circles represent high node support with Bayesian posterior probabilities > 0.95 and maximum bootstrap values > 95. Coloured bars represent hypothesised species groupings

In PCR amplification, the the mitochondrial 12S and three nuclear genes (18S, 28S and NAK) were retrieved with a 96% successful rate, but the mitochondrial COI has a 61.5% failure rate. COI fragments of some samples were not possible to amplify even after repeated optimisations of amplification conditions. We analysed the phylogenetic relationships based on the concatenated datasets of mitochondrial genes (COI and 12S), nuclear genes (18S, 28S and NAK) and all five-gene data (COI, 12S, 18S, 28S and NAK). Although a large proportion of the COI sequences were missing, the divergence of sky-island *Ligidium* species are reflected based on the present mitochondrial genes (Additional file 1). Both Maximum likelihood (ML) and Bayesian (BI) analyses revealed seven main clades [Figs. 4, 5 and 6, maximum bootstrap values (BS) = 100, maximum Bayesian posterior probabilities (PP) = 1]. The support values were high for most nodes in the ML phylogenetic tree and BI consensus tree (Figs. 4, 5, and 6). The ML and BI trees constructed based on the mitochondrial genes represent the same topologies as the phylogenetic trees constructed with the mitochondrial and nuclear genes (Figs. 4, S1 versus Figs. 6, S3). Phylogenetic relationships based on nuclear genes have a different topology, but the same seven clades were retrieved with high support values (Figs. 5a, S2 versus Figs. 4, 6, S1 and S3). The haplotype network (Fig. 5b) shows that the specimens of all molecular operational taxonomic units (MOTUs) have unique haplotypes, not shared with other clusters.

In molecular species delimitation, clade boundaries provided much clearer limits among morphospecies compared to morphology alone. As shown in Fig. 6, all molecular delimitations (ABGD, bPTP, GMYC and BPP) supported the seven-species hypothesis. The seven groups were treated as valid species and are subsequently described below. Moreover, the ambiguous statuses of *L. denticulatum* Shen, 1949, *L. inerme* Nunomura & Xie, 2000 and *L. sichuanense* Nunomura, 2002 in view of morphological characters, are successfully resolved. The minor morphological differences among them were demonstrated to be due to interspecific divergence since the respective clades were well-supported in molecular analyses (Figs. 4, 5 and 6). Further remarks are given in the taxonomic section.

**Fig. 7:**
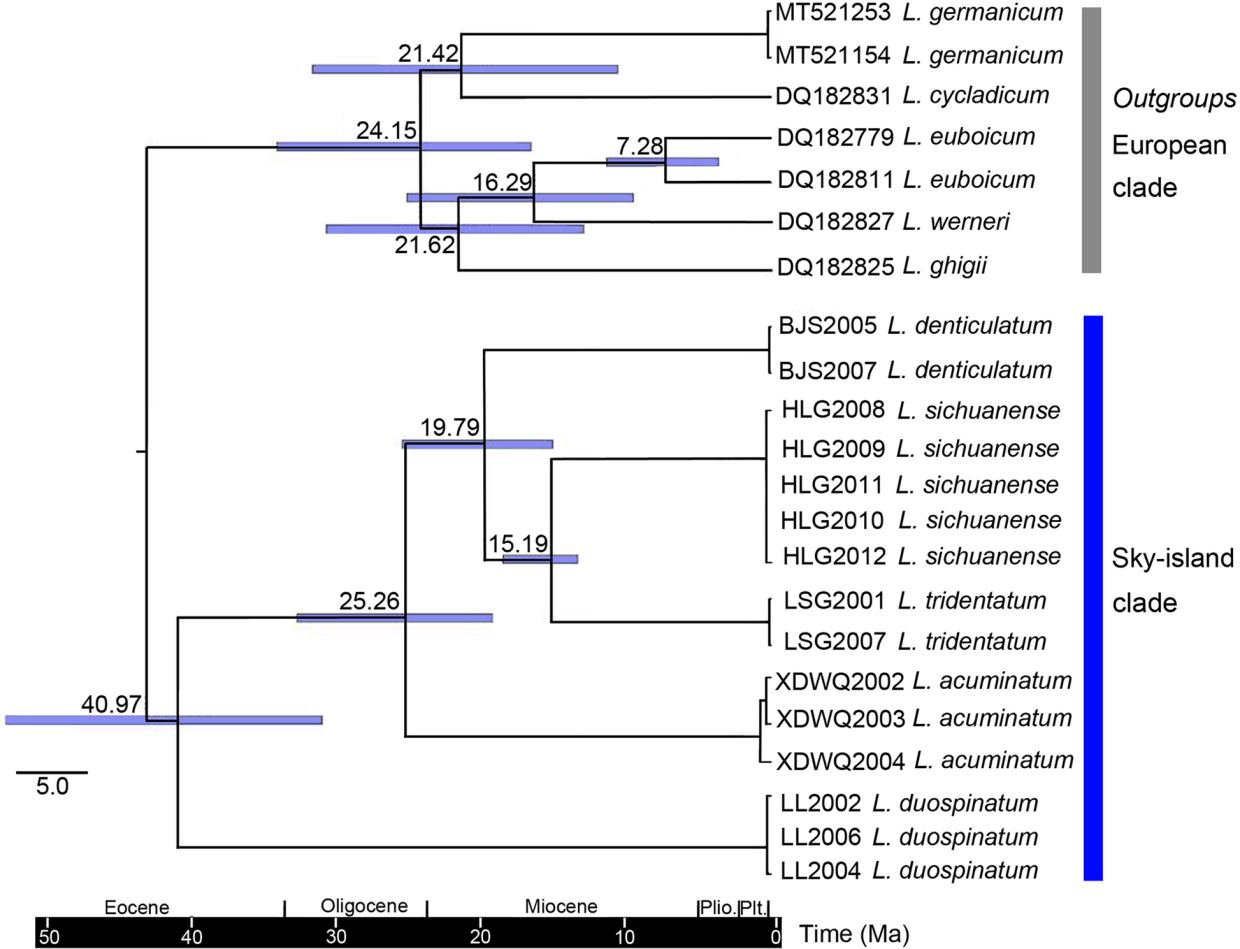
Coalescence tree inferred from BEAST based on COI sequence data. At each node, 95% High Posterior Density intervals and TMRCA (Time to most recent common ancestor) posterior probability densities are shown

Coalescence tree and divergence time estimation indicated that the species divergences within the sky-island clade started in late Eocene (40.97 Mya) to middle Miocene (15.19 Mya), and the earliest divergence event happened in the westernmost site (Lulang, Xizang) (Figs. 2, 7).

### Taxonomy of sky-island *Ligidium* from southwest China

**Fig. 8:**
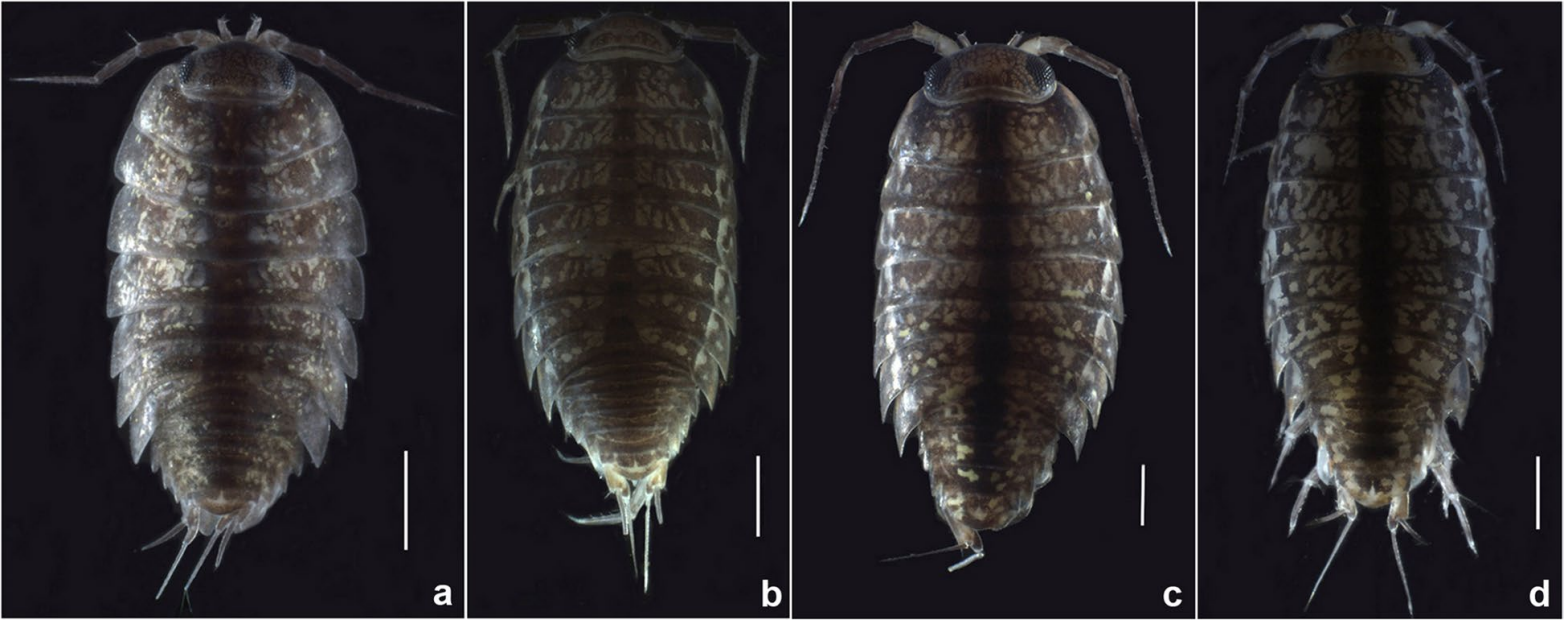
Habitus of *Ligidium* spp. on dorsal view. **a** *L. duospinatum* Li, sp. nov., paratype, female. **b** *L. acuminatum* Li, sp. nov., paratype, male. **c** *L. rotundum* Li, sp. nov., paratype, female. **d** *L. tridentatum* Li, sp. nov., paratype, female. Scale bars: 1 mm

Integrating morphology and molecular analyses, seven sky-island species within the genus *Ligidium* are revealed from southwest China, including four new species (*L. duospinatum* Li, sp. nov., *L. acuminatum* Li, sp. nov., *L. rotundum* Li, sp. nov. and *L. tridentatum* Li, sp. nov.) (Fig. 8). The pereonites 1 and 2 of all the species reported herein lack a “bristle field” on the posterior margin of first pereon-epimera. Descriptions, diagnoses, and illustrations of the new species are provided below.

***Ligidium duospinatum*** **Li, sp. nov.**

(Figs. 8a, 9).

**Fig. 9:**
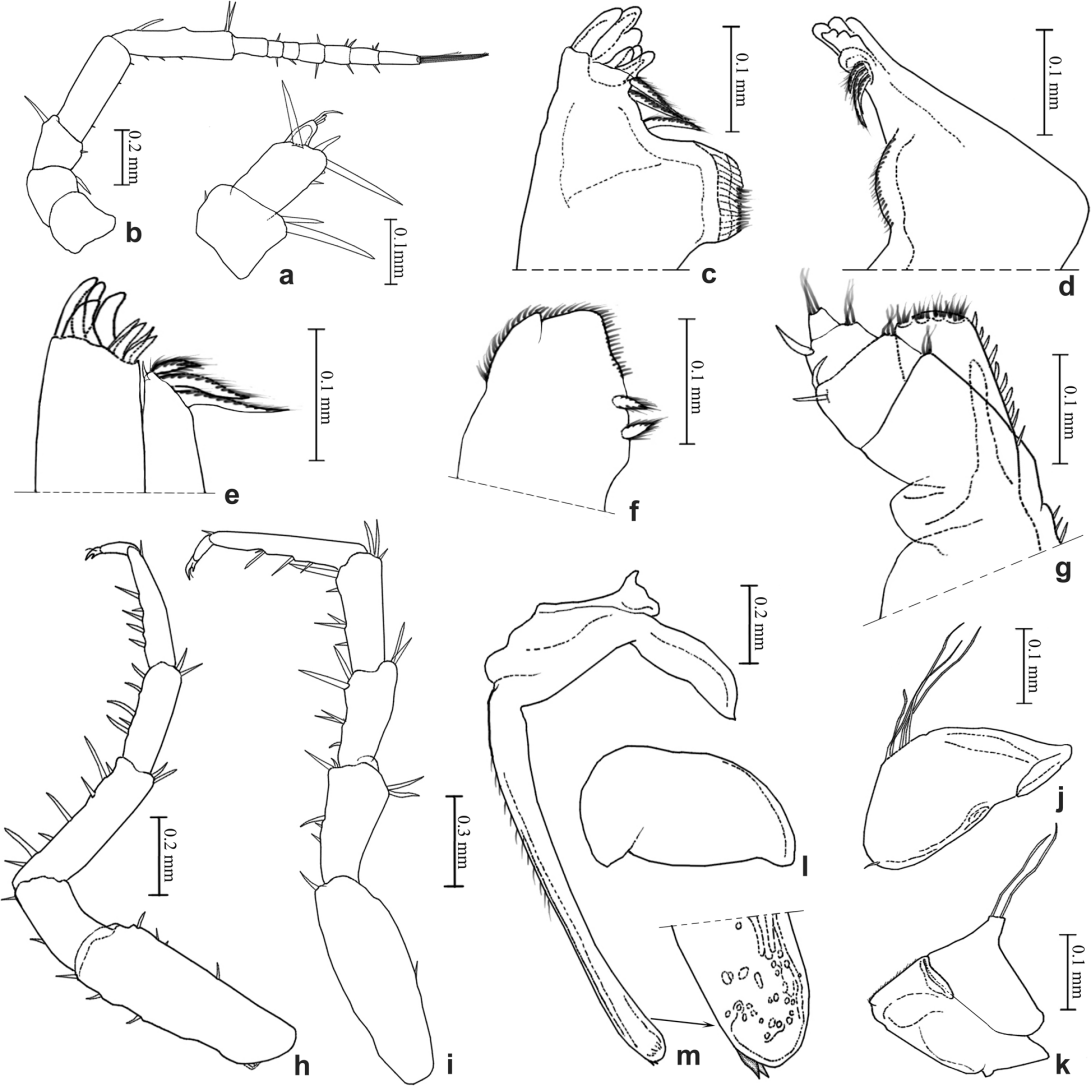
*Ligidium duospinatum* Li, sp. nov., holotype. **a** Antennule. **b** Antenna. **c** Left mandible. **d** Right mandible. **e** Maxillule. **f** Maxilla. **g** Maxilliped. **h** Pereopod 1. **i** Pereopod 7. **j** Pleopod 1 exopodite. **k** Pleopod 1 endopodite. **l** Pleopod 2 exopodite. **m** Pleopod 2 endopodite

### Diagnosis

Unique character of male pleopod 2 endopodite: round apical tip bears 2 small spines on outer margin.

### Type material

Holotype: ♂, China, Xizang (Tibet), Nyingchi, Lulang, Dongbacai Village (29°40′N, 94°44′E), 3496 m a.s.l., 28.vii.2014, leg. W. C. Li (Prep. slides nos. L15164 − 15,185). Paratypes: 16 ♂♂, 39 ♀♀, same data as the holotype (DNA nos. LL2001 − 2004, LL2006; DDBJ accession nos. LC601711 − LC601713, LC602219 − LC602223, LC602572 − LC602576, LC602623 − LC602627, LC637219 − LC637221).

### Etymology

From the Latin prefix *duo-* (= double), and the Latin *spinatus* (= spinous), referring to the male endopodite of pleopod 2 being covered with two spines at apical tip.

### Distribution and habitat

This species is only known from Xizang, China. The type locality is nearby a stream in Dongbacai Village of Lulang Town, southeast Xizang.

### Description

Body length of males 3.5–4.5 mm, of females 3.5–5.0 mm. Colour dark grey with white stigmata and yellowish green spots on dorsal surface (Fig. 8a). Antennule with three small aesthetascs at apex of third article (Fig. 9a). Antenna with flagellum composed of seven articles (Fig. 9b). Left mandible with three-toothed incisor and lacinia mobilis, three penicils between lacinia mobilis and molar process (Fig. 9c); right mandible with three-toothed incisor, lacinia mobilis single toothed, three penicils between lacinia mobilis and molar process (Fig. 9d). Maxillule with inner lobe with three stout penicils and small seta near distal margin (Fig. 9e). Maxilla distally divided into two parts with blunted rounded apices, large lappet with two elongated setose penicils near lateral margin at subapical part (Fig. 9f). Maxilliped with a line of spines on inner margin of endite, palp with three setae on outer margin (Fig. 9g). Pereopod 1 and pereopod 7 without sexual dimorphism (Fig. 9h, i). Pleopod 1 exopodite with rounded distal margin and four long setae, endopodite with triangular projection and two long setae (Fig. 9j, k); male pleopod 2 exopodite similar to broad bean-shaped; endopodite long and thin, apical tip round and bears two small spines on outer margin (Fig. 9l, m).

### Remarks

This new species is similar to *L. inerme* Nunomura & Xie, 2000 in overall appearance, with a relatively broad body with yellowish green spots on the dorsal surface. But it is easily separated from the latter by the flagellum composed of seven articles and the male pleopod 2 endopodite with two small spines at the apical tip (Figs. 9b, m). In *L. inerme*, the flagellum consists of 12 − 15 segments, the male without any denticle at the apical tip of pleopod 2 endopodite [34]. Furthermore, they can also be separated based on molecular delimitation (Figs. 4, 5 and 6). In the analysis of geometric morphometrics, the scatter diagrams indicate that there are differences between them, and the CVAs were able to unambiguously classify the two species with the first two canonical variables (Fig. 3).

***Ligidium acuminatum*** **Li, sp. nov.**

(Figs. 8b, 10).

**Fig. 10:**
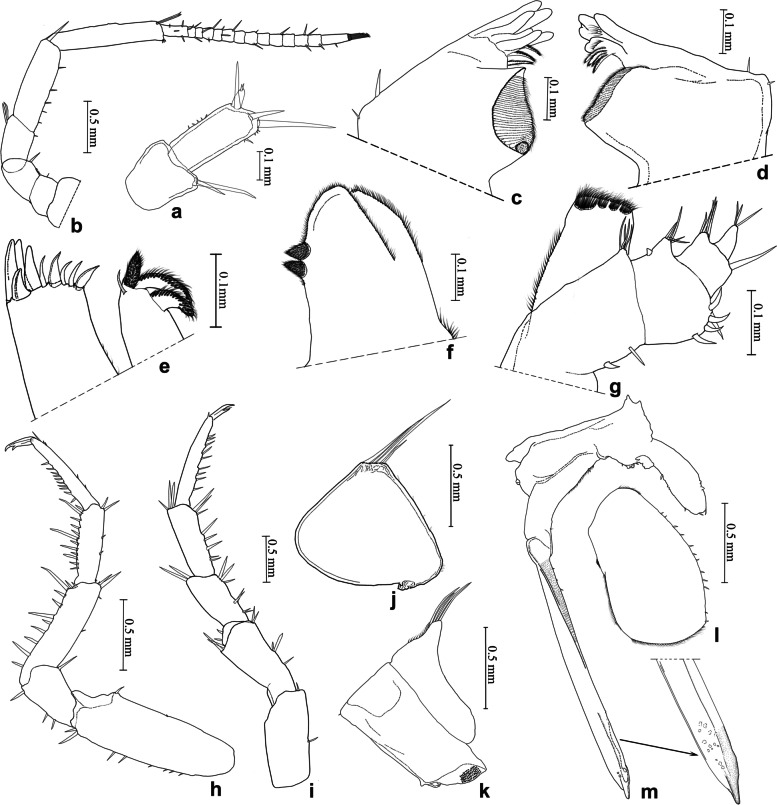
*Ligidium acuminatum* Li, sp. nov. **a** Antennule. **b** Antenna. **c** Left mandible. **d** Right mandible. **e** Maxillule. **f** Maxilla. **g** Maxilliped. **h** Pereopod 1. **i** Pereopod 7. **j** Pleopod 1 exopodite. **k** Pleopod 1 endopodite. **l** Pleopod 2 exopodite. **m** Pleopod 2 endopodite

### Diagnosis

Unique character of male pleopod 2 endopodite: distal part distinctively narrowed towards apical tip without any ornamentations.

### Type material

Holotype: ♂, China, Yunnan Province, Deqin, Xidangwenquan (28°27′N, 98°48′E), 2650 m a.s.l., 28.vii.2014, leg. W. C. Li (Prep. slides nos. L19044 − 19,046). Paratypes: 7 ♂♂, 19 ♀♀, same data as the holotype (DNA nos. XDWQ2001 − 2004; DDBJ accession nos. LC601728 − LC601731, LC602239 − LC602242, LC602592 − LC602595, LC602644 − LC602647, LC637224 − LC637226).

### Etymology

From the Latin *acuminatus* (= acuminate), referring to the male endopod of pleopod 2 ending with an acuminate tip.

### Distribution and habitat

This species is only known from Yunnan Province, China. The specimens collected at an alpine meadow nearby a stream in the northwest and middle Yunnan, respectively.

### Description

Body length of males 6.0 − 7.5 mm, of females 6.0 − 9.0 mm. Colour dark grey with white stigmata on dorsal surface (Fig. 8b). Antennule with two small aesthetascs at the end of third article (Fig. 10a). Antenna with flagellum composed of 14 articles (Fig. 10b). Left mandible with three-toothed incisor and lacinia mobilis, three penicils between lacinia mobilis and molar process (Fig. 10c); right mandible with three-toothed incisor, lacinia mobilis single toothed, four penicils between lacinia mobilis and molar process (Fig. 10d). Maxillule with three stout penicils and long seta on distal margin of inner lobe near (Fig. 10e). Maxilla distally divided into two parts with blunted rounded apices, large lappet with two conical setose penicils near lateral margin at subapical part (Fig. 10f). Maxilliped with 8 setae on outer margin of palp (Fig. 10g). Pereopod 1 and pereopod 7 without sexual dimorphism (Fig. 10h, i). Pleopod 1 exopodite with rounded distal margin and four long setae, endopodite with triangular projection and four long setae (Fig. 10j, k); male pleopod 2 exopodite broad bean-shaped; endopodite long and thin, apical part well-developed sclerotized on inner half, and distinctively narrowed towards acuminate tip (Fig. 10l, m).

### Remarks

**Fig. 11:**
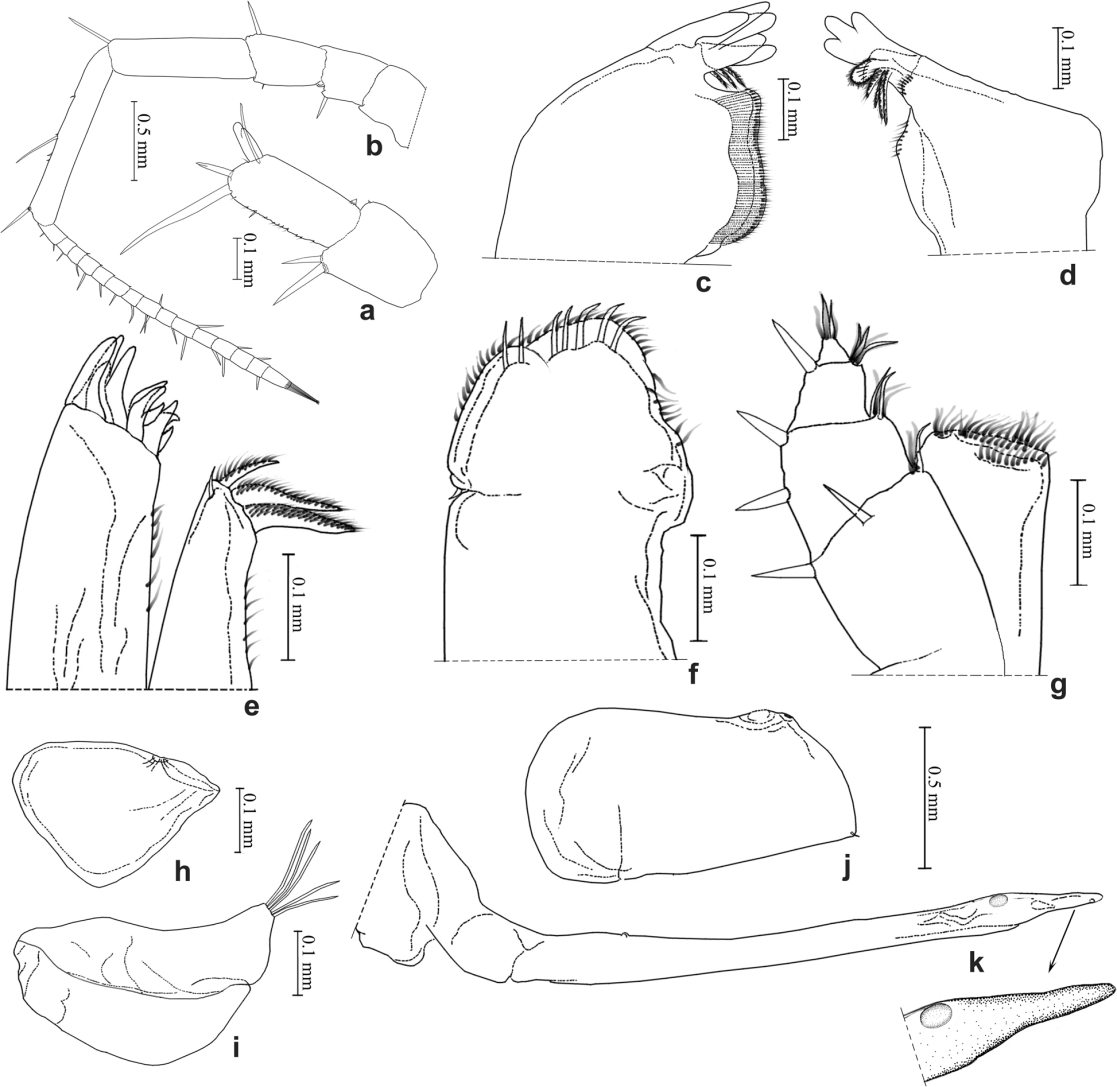
*Ligidium rotundum* Li, sp. nov. **a** Antennule. **b** Antenna. **c** Left mandible. **d** Right mandible. **e** Maxillule. **f** Maxilla. **g** Maxilliped. **h** Pleopod 1 exopodite. **i** Pleopod 1 endopodite. **j** Pleopod 2 exopodite. **k** Pleopod 2 endopodite

This new species is similar to *L. rotundum* Li, sp. nov. in male pleopod 2 endopodite by distal part distinctively narrowed towards the apical tip. But it can be distinguished from the latter one by the male pleopod 2 endopodite without ornamentations (Fig. 10m). In the latter species, pleopod 2 endopodite has a subcircular sclerotized plate at the subapical part (Fig. 11k).

Furthermore, *L. acuminatum* is supported to separate from *L. rotundum* based on molecular analyses (Figs. 4, 5 and 6). In the geometric morphometrics analysis, it is clearly distinguished from the latter species in the CVAs along the first two canonical variables (Fig. 3).

***Ligidium rotundum*** **Li, sp. nov.**

(Figs. 8c, 11).

### Diagnosis

Unique characters of male pleopod 2 endopodite: inner margin with subcircular sclerotized plate at subapical part, distal one sixth conspicuously narrowed towards blunted round apical tip.

### Type material

Holotype: ♂, China, Sichuan Province, Leshan City, Mabian, Mabian Dafengding National Nature Reserve, (28°51’N, 103°31’E), 2100 m a.s.l., 12.viii.2012, leg. W. C. Li and L. Huang (Prep. slides nos. L15042 − 15,053). Paratypes. 1 ♂, 3 ♀♀, with same locality as holotype (DNA nos. DFD2001 − 2003; DDBJ accession nos. LC601697 − LC601699, LC602201 − LC602203, LC602557 − LC602559, LC602608 − LC602610).

### Etymology

From the Latin *rotundus* (= circular), referring to the male endopodite of pleopod 2 being a subcircular sclerotized plate at the subapical part.

### Distribution and habitat

This species is only known from Sichuan Province, China. The type locality is under a waterfall in Mabian Dafengding National Nature Reserve, southwest Sichuan.

### Description

Body length of males 7.0 mm, of females 7.5 − 8.0 mm. Colour dark grey with white mixed with yellowish green stigmata on dorsal surface (Fig. 8c). Antennule with third article gently narrowed towards blunted apical tip (Fig. 11a). Antenna with flagellum composed of 15 articles (Fig. 11b). Left mandible with three-toothed incisor, lacinia mobilis two-toothed, three penicils between lacinia mobilis and molar process (Fig. 11c); right mandible with three-toothed incisor, lacinia mobilis single toothed, four penicils between lacinia mobilis and molar process (Fig. 11d). Maxillule with three stout penicils and small seta near distal margin of inner lobe (Fig. 11e). Maxilla distally divided into two parts with blunted rounded apices, distal margin equipped with seven long setae (Fig. 11f). Maxilliped with four setae on outer margin of palp (Fig. 11g). Pleopod 1 exopodite with rounded distal margin, endopodite with subtriangular projection and five long setae (Fig. 11h, i); male pleopod 2 exopodite similar to broad bean-shaped, endopodite long and thin, and inner margin with subcircular sclerotized plate at subapical part, distal one sixth conspicuously narrowed towards blunted round apical tip (Fig. 11j, k).

### Remarks

No illustration of the male pereopods 1 and 7 are provided since they were missing in collected material.

***Ligidium tridentatum*** **Li, sp. nov.**

(Figs. 8d, 12).

**Fig. 12:**
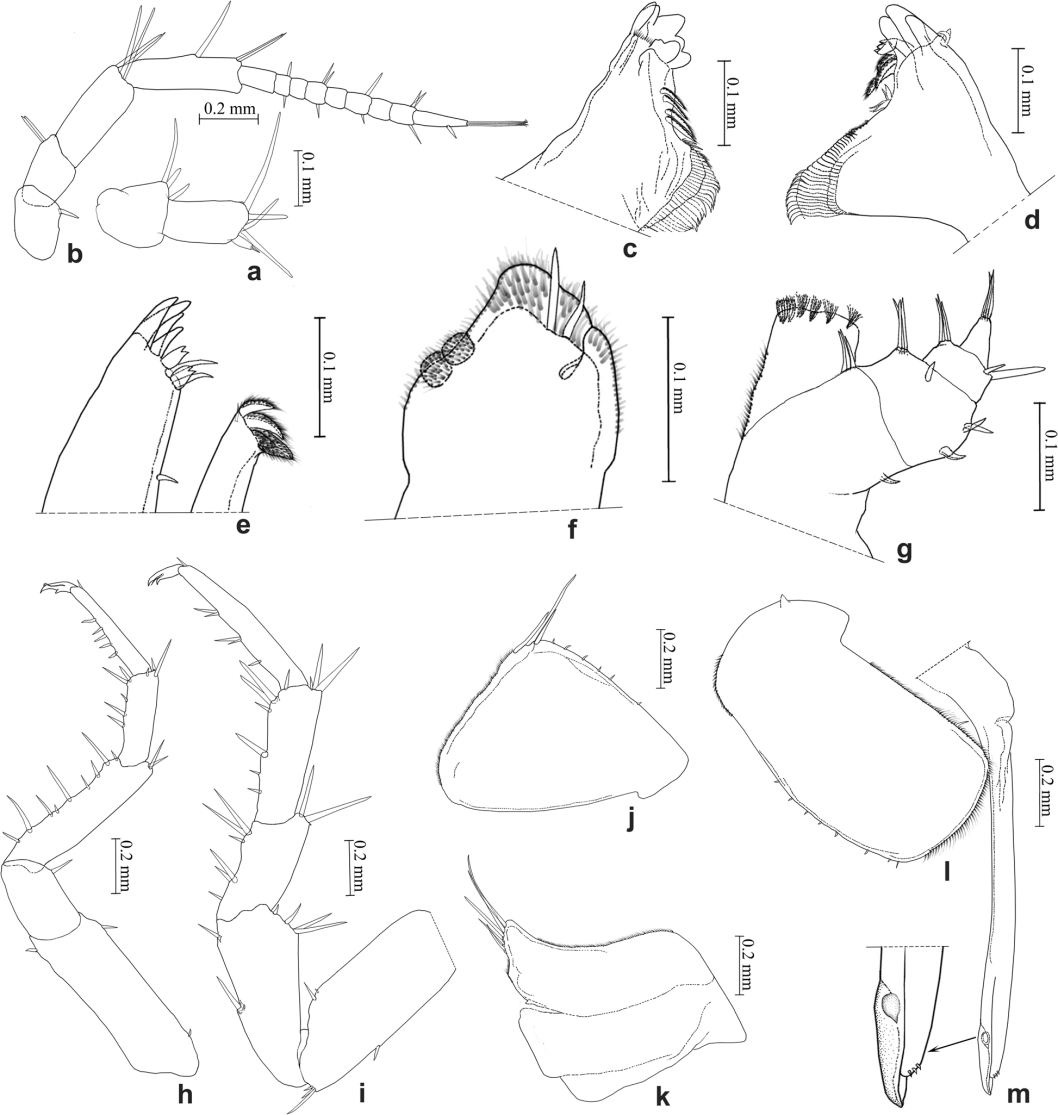
*Ligidium tridentatum* Li, sp. nov. **a** Antennule. **b** Antenna. **c** Left mandible. **d** Right mandible. **e** Maxillule. **f** Maxilla. **g** Maxilliped. **h** Pereopod 1. **i** Pereopod 7. **j** Pleopod 1 exopodite. **k** Pleopod 1 endopodite. **l** Pleopod 2 exopodite. **m** Pleopod 2 endopodite

### Diagnosis

Unique characters of male pleopod 2 endopodite: distal one sixth beak-shaped, inner margin equipped with an almond-shaped projection, and outer margin being three dentations.

### Type material

Holotype: ♂, China, Guizhou Province, Zunyi, Loushanguan (27°59’N, 106°50’E), 1736 m a.s.l., 2.viii.2019, leg. W. C. Li and J. B. Yang (Prep. slides nos. L19029 − 19,033). Paratypes: 7 ♀♀, with same locality as holotype (DNA nos. LSG2001, LSG2004, LSG2006 − 2008; DDBJ accession nos. LC601714 − LC601718, LC602224 − LC602227, LC602577 − LC602581, LC602628 − LC602632, LC637222 − LC637223).

### Etymology

From the Latin *tridentatus* (= tridentate), referring to the male endopodite of pleopod 2 being 3 dentations at the subapical tip.

### Distribution and habitat

This species is only known from Guizhou Province, China. The type locality is under a small bamboo grove near the top of Loushanguan Mount, north Guizhou Province.

### Description

Body length of males 6.0–6.5 mm, of females 4.5–7.0 mm. Colour dark grey with white stigmata on dorsal surface (Fig. 8d). Antennule with 2 aesthetascs at apex of third article (Fig. 12a). Antenna with flagellum composed of nine articles (Fig. 12b). Left mandible with three-toothed incisor and lacinia mobilis, four penicils between lacinia mobilis and molar process (Fig. 12c); right mandible with three-toothed incisor; lacinia mobilis single toothed; three penicils between lacinia mobilis and molar process (Fig. 12d). Maxillule with three stout penicils and small seta near distal margin of inner lobe (Fig. 12e). Maxilla distally inconspicuously divided into two parts with blunted rounded apices, densely covered with hairs and bears two long setae; large lappet with two ovate setose penicils near lateral margin at subapical part (Fig. 12f). Maxilliped with six setae on outer margin of palp (Fig. 12g). Pereopod 1 and pereopod 7 without sexual dimorphism (Fig. 12h, i). Pleopod 1 exopodite with rounded distal margin and three long setae, endopodite with triangular projection and five long setae (Fig. 12j, k); male pleopod 2 exopodite similar to broad bean-shaped; endopodite long and thin, distal one sixth beak-shaped, equipped with almond-shaped projection on inner margin, subapical tip of outer margin being three dentations (Fig. 12l, m).

***Ligidium inerme*** **Nunomura & Xie, 2000**

*Ligidium inerme* Nunomura & Xie, 2000: 44, fig. 1 [34].

### Material examined

1 ♂, 7 ♀♀, **China**: Xizang (Tibet), Nyingchi City, Mêdog, Galongla Snow Mountain (29°44′N, 95°41′E), 3415 m a.s.l., 20 − 23.vii.2014, leg. W. C. Li (Prep. slides nos. L15186 − 15,196; DNA nos. MT2001 − 2004; DDBJ accession nos. LC601724 − LC601727, LC602235 − LC602238, LC602588 − LC602591, LC602640 − LC602643).

### Distribution and habitat

This species has been recorded from Gaoligongshan, Yunnan Province, China [34]. The type locality is the westernmost mount among Hengduan Mountains. Herein, we report this species from the northwesternmost part (Galongla Snow Mountain) of the Hengduan Mountains for the first time. The habitat in which this species has been collected is located at the foot of the south slope of Galongla Snow Mountain. Most parts of the mountain are covered with snow; the vegetation of the collection site is a blend of alpine meadows, shrubs and conifer trees.

### Remarks

The materials examined have a body length of male 6.0 mm and of females 5.5–7.0 mm. Descriptions and illustrations have been adequately given by Nunomura & Xie [34]. The habitus of this species is similar to *L. duospinatum* Li, sp. nov. by having a relatively broad body and dominated with yellowish-green spots on the dorsal surface. It can be distinguished based on the results of comparative morphology, molecular delimitations and geometric morphometrics conducted here, as stated under *L. duospinatum.*

***Ligidium sichuanense*** **Nunomura, 2002**

*Ligidium sichuanense* Nunomura, 2002: 45, figs. 3, 4 [27].

### Material examined

12 ♂♂, 4 ♀♀, **China**: Sichuan, Ganzi, Luding, Hailuogou Glacier (29°36′N, 102°04′E), ∼3000 m a.s.l., 18.viii.2012, leg. W. C. Li and L. Huang (Prep. slide nos. L15068 − L15081, L20141 − 20,160, L20165 − L20166; DNA nos. HLG2007 − 2012; DDBJ accession nos. LC601705 − LC601710, LC602213 − LC602218, LC602566 − LC602571, LC602617 − LC602622, LC637214 − LC637218).

### Distribution and habitat

The type locality of this species is located at Chapingshan (alt. 3820 m), Mao County, Sichuan Province, China [27]. Herein, we report it from the monsoon glacier area of western Sichuan Province, China. The habitat in which this species has been collected is as same as *L. glacialis* Li, sp. nov.

### Remarks

The materials examined have a body length of males 4.0–6.0 mm and of females 5.5–7.0 mm. Descriptions and illustrations of this species have been adequately provided by Nunomura [27]. This species is allied to *L. denticulatum* Shen, 1949 and *L. inerme*, with the morphological differences among them having been given by Nunomura [27]. We provided additional molecular and geometric morphometrics evidence to distinguish them. Every species was represented by a well-supported clade (Figs. 4, 5 and 6) and an unambiguous classification along the first two canonical variables (Fig. 3).

***Ligidium denticulatum*** **Shen, 1949**

*Ligidium denticulatum* Shen, 1949: 50, fig. A1–14 [71]; Kwon & Taiti, 1993: 6, figs. 13–26 [31]; Nunomura & Xie, 2000: 46, fig.  2 [34].

### Material examined

29 ♂♂, 72 ♀♀, **China**: Guizhou Province, Liupanshui City, Bijiashan Park, 26°35’N, 104°53’E, 1821 m a.s.l., 9.viii.2019, leg. W. C. Li and J. B. Yang (Prep. slides nos. L19025 − L19028; DNA nos. BJS2001 − 2010, BJS2012; DDBJ accession nos. LC601687 − LC601696, LC602190 − LC602200, LC602545 − LC602556, LC602596 − LC602607; LC637212 − LC637213).

### Distribution and habitat

Shen originally described this species from Kunming Lake, Yunnan Province, China in 1949 [71]; afterwards, it was also illustrated based on specimens from Kunming [31, 34]. Herein, we report it from Guizhou Province, China, for the first time. The samples examined were collected at Bijiashan Park and Minghu National Wetland Park, west Guizhou Province, China. The soil at Bijiashan Park has high moisture, and the vegetation of the collection site is composed of evergreen broad-leaf trees; the Minghu National Wetland Park mainly consists of meadow, evergreen shrubs and a lake.

### Remarks

The materials examined have a body length of males 4.0–6.5 mm and of females 3.0–9.0 mm. Descriptions and illustrations have been adequately given in [31, 34, 71]. This species is similar to *L. sichuanense* Nunomura, 2002 and *L. inerme* Nunomura & Xie, 2000. Nunomura has mentioned the morphological differences among them [34]. Herein, we provided additional molecular and geometric morphometrics evidence to distinguish them, as stated under *L. sichuanense*.



## Conclusion and discussion

In terrestrial Isopod taxonomy, different genera usually exhibit several morphological characters useful in species diagnoses, such as in male pereopods and pleopod 1 exopods or even characters of pereonites and pleonites. In classical morphological taxonomy of species in the genus *Ligidium*, the male pleopod 2 endopodites provide the most significant diagnostic character, especially its apical part showing a considerable interspecific difference in shape [13, 14]. However, morphological boundaries among species of terrestrial isopods are still ambiguous. In addition, minor interspecific differences among certain congeners and the varying extent of intraspecific variation create a lot of confusion in species recognition. As early as 1923, Jackson had mentioned that morphological variation should not be underestimated in species identification [15]. To resolve these issues, the integration of morphology and molecular data has been considered an effective way to evaluate current taxonomy and explore possible existence of new species before describing them [14, 72–77]. Most integrative taxonomy studies on terrestrial isopods combine molecular species delimitation methods with a traditional qualitative morphological approach, but it is necessary to apply a standardized investigation on morphology.

Geometric morphometrics involves using standardized images and landmarks to visualize and test for differences in shape between samples, which has been applied effectively to aid species delimitation [78]. Until today, the geometric morphometrics technique has been applied to detect body-shape variation among individuals of the same terrestrial isopod species in two different species [79–81], but it remains questionable whether it would work in species identification of terrestrial isopods. Herein, we evaluate *Ligidium* species variation in body shape using two-D landmarks data. Results show that the morphospace was distinctly separated among the species in CVA, but it overlaps extensively in PCA (Fig. 3). This could be attributed to the properties of the different multivariate statistical techniques. In PCA, linear combinations of the original variables are derived which explain the maximum amount of variation in the data set and which are orthogonal. These principal components summarize the data with as little loss of information as possible [82]. In contrast, linear combinations of the original variables are selected to maximize the ratio of between-sample to within-sample variance in CVA. The canonical variates are not necessarily orthogonal and the actual angle between the canonical variates can be calculated [82]. Our study demonstrated that CVA is superior to PCA in exploring morphological variation in *Ligidium*.

In integrative taxonomy of terrestrial isopods, molecular data have been showed to be effective in resolving morphological taxonomy ambiguities. Despite the undeniable importance of classical taxonomy in species discovery, molecular data are becoming an integrative part of taxonomic practice [14, 72–77]. In molecular analyses of *Ligidium* species, the use of mitochondrial fragments in association with morphology has helped to alleviate the taxonomic impediment through the discovery, delimitation and description of new species [14, 73]. Nevertheless, the mitochondrial data have been proposed to limited applicability for reconstructing the phylogeny of Isopoda [83]. Thus, we established phylogenetic relationships of the sky-island *Ligidium* species in southwest China using two mitochondrial genes (COI and 12S), and the concatenated mitochondrial and nuclear data (COI, 12S, 18S, 28S and NAK), respectively. The mitochondrial gene trees not only represent the same topologies but also show the clades with high support values as the latter concatenated gene trees (Figs. 4, S1 versus Figs. 6, S3). These results indicated that the mitochondrial genes should be applied effectively to aid species delimitation in the sky-island *Ligidium*. Furthermore, the statistical parsimony haplotype network (Fig. 5b) based on nuclear genes shows that the specimens of all MOTUs have unique haplotypes. These results indicate that nuclear genes, if properly selected, can be considered as additional DNA evidence in terrestrial isopod taxonomy.

We revealed seven sky-island *Ligidium* species by integrating comparative morphology, geometric morphometrics and molecular analysis. Problems due to minor interspecific morphological differences were resolved by integrative taxonomy. Furthermore, we estimated the divergence time of sky-island *Ligidium* in southwest China. The timeframe of cladogenetic events coincides with the uplift of the Qinghai-Tibetan Plateau. The estimation indicated that the divergence of sky-island *Ligidium* species started in the late Eocene to the middle Miocene (Fig. 7), probably driven by the massive uplift of Qinghai-Tibetan Plateau. The tectonic activity changed the topography as well as heightened the environmental heterogeneity of the Qinghai-Tibetan Plateau and its adjacent areas [84, 85]. The extremely complex topography of southwest China can probably restrict the dispersal of the sky-island *Ligidium* species. Species distributed in different mountainous areas may have evolved independently, resulting in phenotypic divergence and genetic differentiation. In addition, the high environmental heterogeneity promotes species richness, affecting species richness of terrestrial isopods both directly and indirectly [86]. Thus, we deduced that tectonic activity in the late Eocene and the middle Miocene is one of the principal reasons for species divergence and high species richness of the sky-island *Ligidium* in southwest China. To date, the occurrence of sky-island *Ligidium* species in southwest China shows many distribution gaps (Fig. 2). According to our field experience, most mountainous areas of southwest China have similar habitats and are suitable for *Ligidium*. Hence, the mountainous habitats of southwest China may hide an unexpected wealth of sky-island *Ligidium* species.

In conclusion, the integrative taxonomical approach is essential to enlighten the sky-island *Ligidium* taxonomy. We demonstrated that the pleopod 2 endopodite is a reliable diagnostic character for this group of species. The addition of morphometrics and molecular analyses is needed to delimit species in a more precise and secure way. Our estimation of divergence time among sky-island *Ligidium* species showed the uplift of Qinghai-Tibetan Plateau between the late Eocene and the middle Miocene to be one of the principal factors in sky-island *Ligidium* species divergence and high species richness in southwest China. We inferred also that sky-island mountains most probably are a huge reserve of *Ligidium* species diversity.

## Supplementary Information

**Additional file 1.** Description of the molecular sequences were used for phylogenetic analysis, including DNAnumber, taxon, collection locality and DDBJ/NCBI accession number.

**Additional file 2.** Overview of localitieswhere *Ligidium* species have been recordedin this context.

**Additional file 3.** Supplementary figures of maximum likelihood treesbased on two mitochondrial genes (COI and 12S), three nuclear genes (18S, 28Sand NAK) and a concatenated dataset of five loci (COI, 12S, 18S, 28S and NAK).

## Data Availability

The gene sequences of *Ligidium* species are deposited in the DNA Data Bank of Japan (DDBJ) under the accession numbers included in supplementary information additional file 1. All *Ligidium* specimens in this context are conserved in absolute ethanol in Insect Museum, Jiangxi Agricultural University, Nanchang, China (JXAUM).

## Abbreviations

JXAUM: Insect Museum, Jiangxi Agricultural University, Nanchang, China
COI: Cytochrome c oxidase I
NAK: Nuclear protein-coding genes Sodium-otassium Pump
PCR: Polymerase chain reaction
DDBJ: DNA Data Bank of Japan
ML: Maximum likelihood
BI: Bayesian inference
MCMC: Markov chain Monte Carlo
PP: Posterior probability
ABGD: Automatic barcode gap discovery
BPP: Bayesian species delimitation
bPTP: Bayesian implementation of the Poisson tree processes model for species delimitation
GMYC: General mixed Yule coalescent
PCAs: Principal component analyses
CVAs: Canonical variate analyses
TMRCA: Time to most recent common ancestor
