# Supplementary material for: Integrative taxonomy on the rare sky-island Ligidium species from southwest China (Isopoda, Oniscidea, Ligiidae)
Source: BMC Zool. 2022 May 23;7:26. doi: 10.1186/s40850-022-00120-1 (PMC10127345; doi:10.1186/s40850-022-00120-1)
Supplement: Supplementary file 1 — Additional file 1. Description of the molecular sequences were used for phylogenetic analysis, including DNAnumber, taxon, collection locality and DDBJ/NCBI accession number. [file 40850_2022_120_MOESM1_ESM.docx]

**Integrative taxonomy** **on the rare sky-island** ***Ligidium*** **species from southwest China (Isopoda, Oniscidea, Ligiidae)**

Jin Wang^1^, Jingbo Yang^1^, Xuegang Zeng^1^ and Weichun Li^1^

1 College of Agronomy, Jiangxi Agricultural University, Nanchang 330045, China

Corresponding author: Weichun Li ([weichunlee@126.com](mailto:weichunlee@126.com))

**Additional file 1.** Description of the molecular sequences were used for phylogenetic analysis, including DNA number, taxon, collection locality and DDBJ/NCBI accession number.

| DNA no. | Taxon | Collection locality | COI | 12S | 18S | 28S | NAK |
| --- | --- | --- | --- | --- | --- | --- | --- |
| BJS2001 | *L.* *denticulatum* | China, Guizhou, Liupanshui, Bijiashan | - | LC602190 | LC602545 | LC602596 | LC601687 |
| BJS2002 | *L.* *denticulatum* | China, Guizhou, Liupanshui, Bijiashan | - | LC602191 | LC602546 | LC602597 | LC601688 |
| BJS2003 | *L.* *denticulatum* | China, Guizhou, Liupanshui, Bijiashan | - | LC602192 | LC602547 | LC602598 | LC601689 |
| BJS2004 | *L.* *denticulatum* | China, Guizhou, Liupanshui, Bijiashan | - | LC602193 | LC602548 | LC602599 | LC601690 |
| BJS2005 | *L.* *denticulatum* | China, Guizhou, Liupanshui, Bijiashan | LC637212 | LC602194 | LC602549 | LC602600 | LC601691 |
| BJS2006 | *L.* *denticulatum* | China, Guizhou, Liupanshui, Bijiashan | - | LC602195 | LC602550 | LC602601 | LC601692 |
| BJS2007 | *L.* *denticulatum* | China, Guizhou, Liupanshui, Bijiashan | LC637213 | LC602196 | LC602551 | LC602602 | LC601693 |
| BJS2008 | *L.* *denticulatum* | China, Guizhou, Liupanshui, Bijiashan | - | LC602197 | LC602552 | LC602603 | LC601694 |
| BJS2009 | *L.* *denticulatum* | China, Guizhou, Liupanshui, Bijiashan | - | LC602198 | LC602553 | LC602604 | LC601695 |
| BJS2010 | *L.* *denticulatum* | China, Guizhou, Liupanshui, Bijiashan | - | LC602199 | LC602554 | LC602605 | LC601696 |
| BJS2011 | *L.* *denticulatum* | China, Guizhou, Liupanshui, Bijiashan | - | - | LC602555 | LC602606 | - |
| BJS2012 | *L.* *denticulatum* | China, Guizhou, Liupanshui, Bijiashan | - | LC602200 | LC602556 | LC602607 | - |
| DFD2001 | *L.* *rotundum* | China, Sichuan, Mabian, Dafengding | - | LC602201 | LC602557 | LC602608 | LC601697 |
| DFD2002 | *L.* *rotundum* | China, Sichuan, Mabian, Dafengding | - | LC602202 | LC602558 | LC602609 | LC601698 |
| DFD2003 | *L.* *rotundum* | China, Sichuan, Mabian, Dafengding | - | LC602203 | LC602559 | LC602610 | LC601699 |
| HLG2007 | *L.* *sichuanense* | China, Sichuan, Ganzi, Hailuogou Glacier | - | LC602213 | LC602566 | LC602617 | LC601705 |
| HLG2008 | *L.* *sichuanense* | China, Sichuan, Ganzi, Hailuogou Glacier | LC637214 | LC602214 | LC602567 | LC602618 | LC601706 |
| HLG2009 | *L.* *sichuanense* | China, Sichuan, Ganzi, Hailuogou Glacier | LC637215 | LC602215 | LC602568 | LC602619 | LC601707 |
| HLG2010 | *L.* *sichuanense* | China, Sichuan, Ganzi, Hailuogou Glacier | LC637216 | LC602216 | LC602569 | LC602620 | LC601708 |
| HLG2011 | *L.* *sichuanense* | China, Sichuan, Ganzi, Hailuogou Glacier | LC637217 | LC602217 | LC602570 | LC602621 | LC601709 |
| HLG2012 | *L.* *sichuanense* | China, Sichuan, Ganzi, Hailuogou Glacier | LC637218 | LC602218 | LC602571 | LC602622 | LC601710 |
| LL2001 | *L.* *duospinatum* | China, Tibet, Lulang, Dongbacai Village | - | LC602219 | LC602572 | LC602623 | LC601711 |
| LL2002 | *L.* *duospinatum* | China, Tibet, Lulang, Dongbacai Village | LC637219 | LC602220 | LC602573 | LC602624 | LC601712 |
| LL2003 | *L.* *duospinatum* | China, Tibet, Lulang, Dongbacai Village | - | LC602221 | LC602574 | LC602625 | LC601713 |
| LL2004 | *L.* *duospinatum* | China, Tibet, Lulang, Dongbacai Village | LC637220 | LC602222 | LC602575 | LC602626 | - |
| LL2006 | *L.* *duospinatum* | China, Tibet, Lulang, Dongbacai Village | LC637221 | LC602223 | LC602576 | LC602627 | - |
| LSG2001 | *L.* *tridentatum* | China, Guizhou, Zunyi, Loushanguan | LC637222 | LC602224 | LC602577 | LC602628 | LC601714 |
| LSG2004 | *L.* *tridentatum* | China, Guizhou, Zunyi, Loushanguan | - | - | LC602578 | LC602629 | LC601715 |
| LSG2006 | *L.* *tridentatum* | China, Guizhou, Zunyi, Loushanguan | - | LC602225 | LC602579 | LC602630 | LC601716 |
| LSG2007 | *L.* *tridentatum* | China, Guizhou, Zunyi, Loushanguan | LC637223 | LC602226 | LC602580 | LC602631 | LC601717 |
| LSG2008 | *L.* *tridentatum* | China, Guizhou, Zunyi, Loushanguan | - | LC602227 | LC602581 | LC602632 | LC601718 |
| MT2001 | *L.* *inerme* | China, Tibet, Mêdog, Galongla Snow Mountain | - | LC602235 | LC602588 | LC602640 | LC601724 |
| MT2002 | *L.* *inerme* | China, Tibet, Mêdog, Galongla Snow Mountain | - | LC602236 | LC602589 | LC602641 | LC601725 |
| MT2003 | *L.* *inerme* | China, Tibet, Mêdog, Galongla Snow Mountain | - | LC602237 | LC602590 | LC602642 | LC601726 |
| MT2004 | *L.* *inerme* | China, Tibet, Mêdog, Galongla Snow Mountain | - | LC602238 | LC602591 | LC602643 | LC601727 |
| XDWQ2001 | *L.* *acuminatum* | China, Yunnan, Deqin, Xidangwenquan | - | LC602239 | LC602592 | LC602644 | LC601728 |
| XDWQ2002 | *L.* *acuminatum* | China, Yunnan, Deqin, Xidangwenquan | LC637224 | LC602240 | LC602593 | LC602645 | LC601729 |
| XDWQ2003 | *L.* *acuminatum* | China, Yunnan, Deqin, Xidangwenquan | LC637225 | LC602241 | LC602594 | LC602646 | LC601730 |
| XDWQ2004 | *L.* *acuminatum* | China, Yunnan, Deqin, Xidangwenquan | LC637226 | LC602242 | LC602595 | LC602647 | LC601731 |
| - | *L.* *cycladicum* | Greek | DQ182831 | - | - | - | - |
| - | *L. euboicum* | Greek | DQ182779 | - | - | - | - |
| - | *L. euboicum* | Greek | DQ182811 | - | - | - | - |
| - | *L. germanicum* | Germany, Bavaria | MT521154 | - | - | - | - |
| - | *L. germanicum* | Germany, Bavaria | MT521253 | - | - | - | - |
| - | *L. ghigii* | Greek | DQ182825 | - | - | - | - |
| - | *L. werneri* | Greek | DQ182827 | - | - | - | - |
| - | *Armadillidium vulgare* | - | MH279710 | AF259522 | KR424675 | AY739196 | - |
| - | *Porcellionides pruinosus* | - | KR424606 | KX467638 | KR424622 | MG888010 | MN234275 |
| - | *Styloniscus* sp. | - | KR424587 | KX467641 | KR424679 | KR424725 | - |
| - | *Spherillo dorsalis* | - | AB861897 | AB861905 | LC027528 | AB861925 | - |
| - | *Spherillo obscurus* | - | LC496525 | AB861907 | AB861921 | LC496526 | - |

DDBJ accession numbers are given in separate columns for each gene.
