# Supplementary material for: Integrative taxonomy on the rare sky-island Ligidium species from southwest China (Isopoda, Oniscidea, Ligiidae)
Source: BMC Zool. 2022 May 23;7:26. doi: 10.1186/s40850-022-00120-1 (PMC10127345; doi:10.1186/s40850-022-00120-1)
Supplement: Supplementary file 2 — Additional file 2. Overview of localitieswhere Ligidium species have been recordedin this context. [file 40850_2022_120_MOESM2_ESM.docx]

**Integrative taxonomy** **on the rare sky-island** ***Ligidium*** **species from southwest China (Isopoda, Oniscidea, Ligiidae)**

Jin Wang^1^, Jingbo Yang^1^, Xuegang Zeng^1^ and Weichun Li^1^

1 College of Agronomy, Jiangxi Agricultural University, Nanchang 330045, China

Corresponding author: Weichun Li ([weichunlee@126.com](mailto:weichunlee@126.com))

**Additional file 2.** Overview of localities where *Ligidium* species have been recorded in this context.

| Taxa | Collection locality | Longitude | Latitude |
| --- | --- | --- | --- |
| *L. acuminatum* **sp. nov.** | **Yunnan**, Xidang, Wenquan, alt. 2650 m | 98.8000 | 28.4500 |
|  | Yuxi, Mopanshan, alt. 2102 m | 101.9399 | 23.9588 |
| *L. denticulatum* | **Yunnan**, Kunming, alt. 2200 m (Kwon and Taiti 1993) | 102.7050 | 25.0417 |
|  | **Guizhou**, Liupanshui, Bijiashan, alt. 1821 m | 104.8906 | 26.5831 |
|  | Liupanshui, Minghu National Wetland Park, alt. 1806 m | 104.8087 | 26.5786 |
| *L. duospinatum* **sp. nov.** | **Tibet**, Lulang, Dongbacai Village, alt. 3496 m | 94.7356 | 29.6737 |
| *L. inerme* | **Yunnan**, Tengchong, Gaoligongshan, alt. 2280−2290 m (Nunomura and Xie 2000) | 98.7000 | 27.1167 |
|  | Baoshan, Baihualing, alt. 2100−2550 m (Nunomura and Xie 2000) | 99.1667 | 25.1333 |
|  | **Tibet**, Mêdog, Galongla Snow Mountain, alt. 3415 m | 95.6768 | 29.7382 |
| *L. rotundum* **sp. nov.** | **Sichuan**, Mabian, Mabian Dafengding National Nature Reserve, alt. 2100 m | 103.5167 | 28.8500 |
| *L. sichuanense* | **Sichuan**, Mao County, Chapingshan, alt. 3820 m (Nunomura 2002) | 103.8333 | 31.5000 |
|  | Ganzi, Hailuogou Glacier, near no. 1 glacier, alt. 3000 m | 102.0667 | 29.6000 |
| *L. tridentatum* **sp. nov.** | **Guizhou**, Zunyi, Loushanguan, alt. 1736 m | 106.8436 | 27.9982 |

The Chinese province names are given in bold and geographical coordinates in decimal system.
