# Supplementary material for: Integrative taxonomy on the rare sky-island Ligidium species from southwest China (Isopoda, Oniscidea, Ligiidae)
Source: BMC Zool. 2022 May 23;7:26. doi: 10.1186/s40850-022-00120-1 (PMC10127345; doi:10.1186/s40850-022-00120-1)
Supplement: Supplementary file 3 — Additional file 3. Supplementary figures of maximum likelihood treesbased on two mitochondrial genes (COI and 12S), three nuclear genes (18S, 28Sand NAK) and a concatenated dataset of five loci (COI, 12S, 18S, 28S and NAK). [file 40850_2022_120_MOESM3_ESM.docx]

**Integrative taxonomy** **on the rare sky-island** ***Ligidium*** **species from southwest China (Isopoda, Oniscidea, Ligiidae)**

Jin Wang^1^, Jingbo Yang^1^, Xuegang Zeng^1^ and Weichun Li^1^

1 College of Agronomy, Jiangxi Agricultural University, Nanchang 330045, China

Corresponding author: Weichun Li ([weichunlee@126.com](mailto:weichunlee@126.com))

**Additional file 3.** Supplementary figures of maximum likelihood trees based on two mitochondrial genes (COI and 12S), three nuclear genes (18S, 28S and NAK) and a concatenated dataset of five loci (COI, 12S, 18S, 28S and NAK).


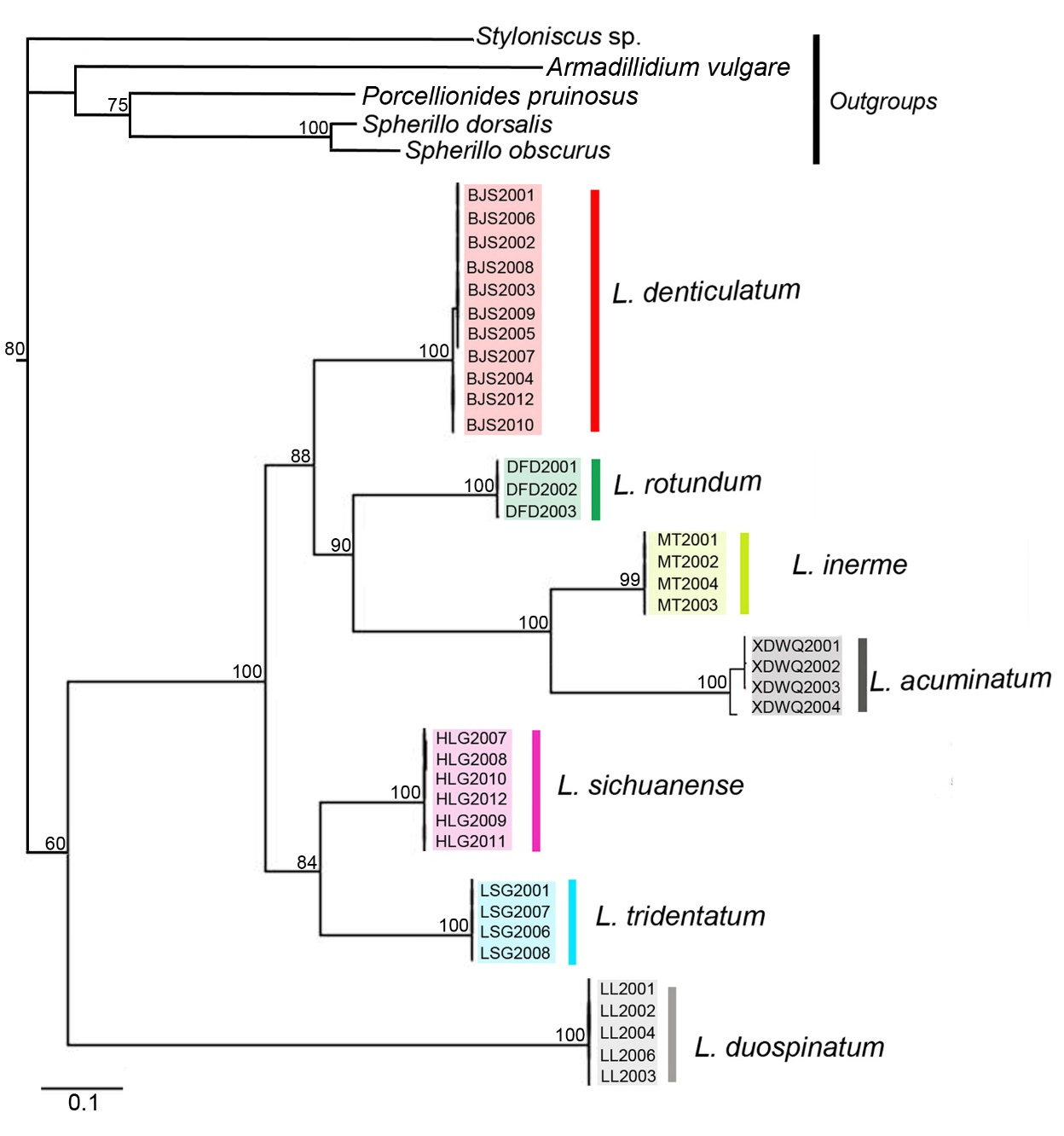


**Fig. S1** Maximum likelihood tree based on a concatenated dataset of two mitochondrial genes (COI and 12S). Numbers on nodes represent bootstrap values.


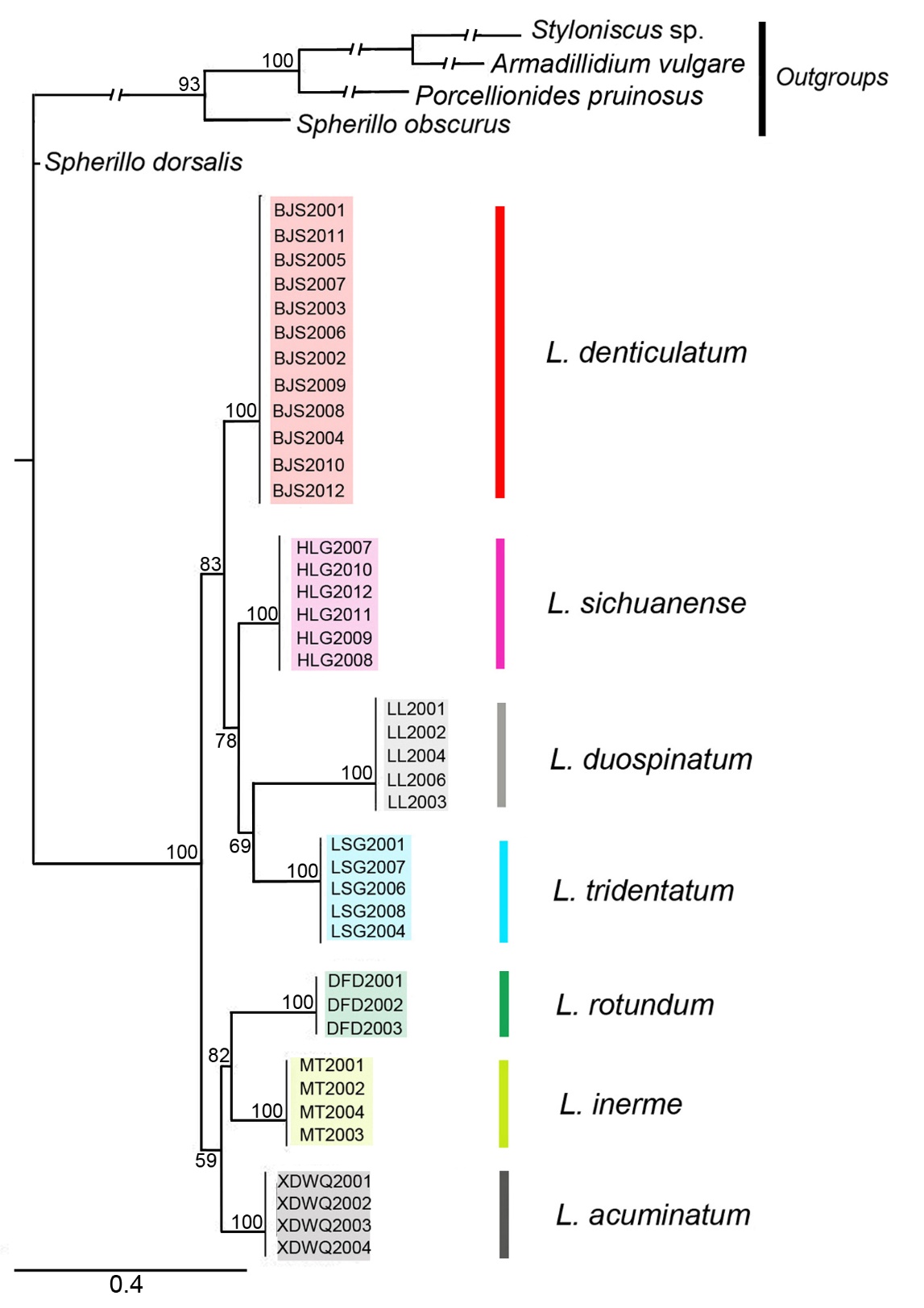


**Fig. S2** Maximum likelihood tree based on a concatenated dataset of three nuclear genes (18S, 28S and NAK). Numbers on the nodes represent bootstrap values.


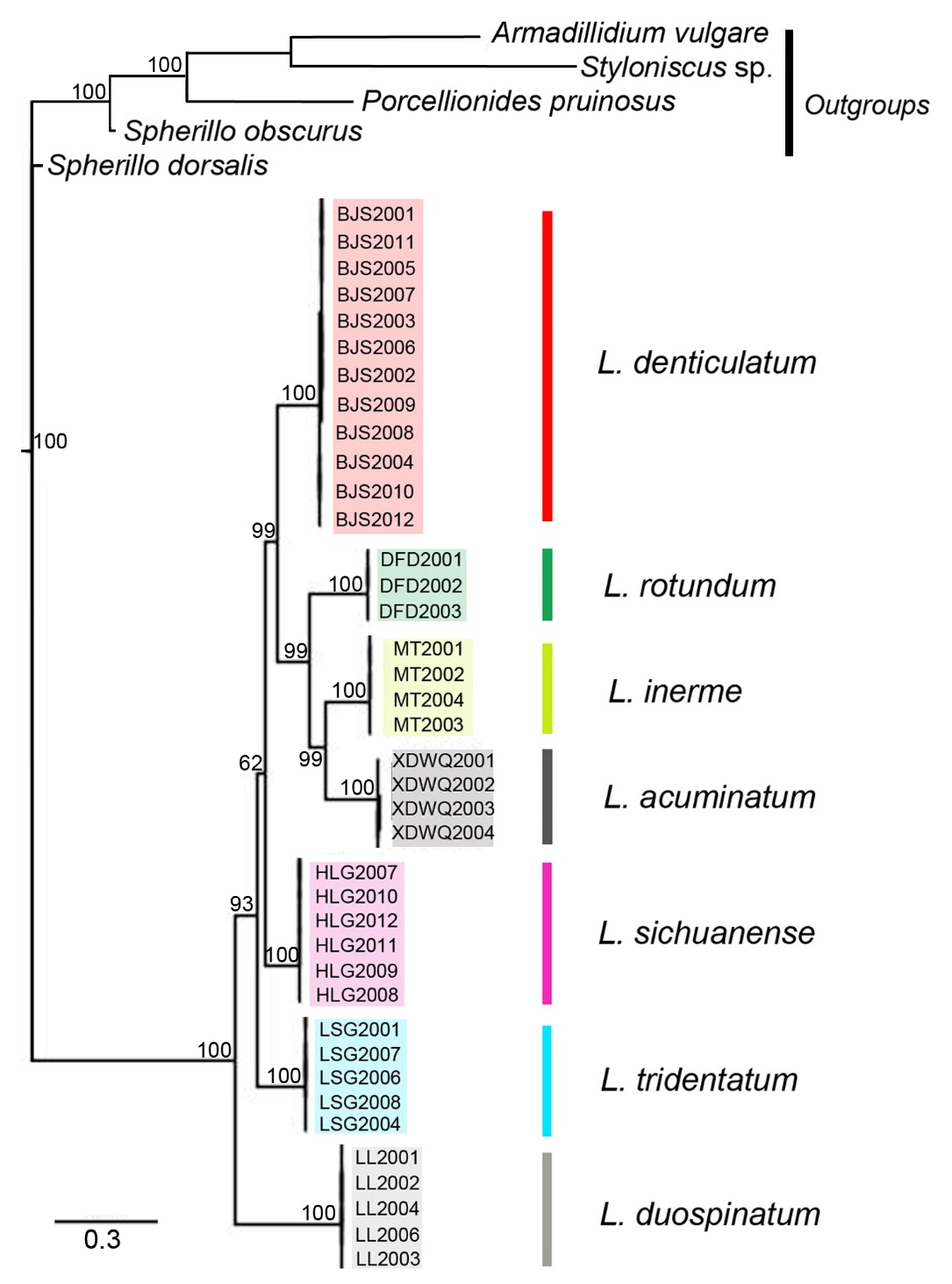


**Fig. S3** Maximum likelihood tree based on a concatenated dataset of five loci (COI, 12S, 18S, 28S and NAK). Numbers on nodes represent bootstrap values.
